# Policies, projections, and the social cost of carbon: Results from the DICE-2023 model

**DOI:** 10.1073/pnas.2312030121

**Published:** 2024-03-19

**Authors:** Lint Barrage, William Nordhaus

**Affiliations:** ^a^Department of Management, Technology, and Economics, ETH Zurich, Zurich 8092, Switzerland; ^b^Department of Economics, Yale University, New Haven, CT 06520; ^c^National Bureau of Economic Research, Cambridge, MA 02138

**Keywords:** DICE model, economics of climate change, social cost of carbon, integrated assessment

## Abstract

The present study examines the assumptions, modeling structure, and results of DICE-2023, the revised Dynamic Integrated Model of Climate and the Economy (DICE), updated to 2023. The revision contains major changes in the treatment of risk, the carbon and climate modules, the treatment of nonindustrial greenhouse gases, discount rates, as well as updates on all the major components. Noteworthy changes are a significant reduction in the target for the optimal (cost-beneficial) temperature path, a lower cost of reaching the 2 °C target, an analysis of the impact of the Paris Accord, and a major increase in the estimated social cost of carbon.

## Significance

The DICE model is the most widely used climate-change integrated assessment model, employed in calculating the social cost of carbon by the US and other governments as well as for creating consistent scenarios and evaluating policies and uncertainties. The present study updates the 2016 DICE version and introduces approaches for including nondiversifiable risk, includes a revised carbon cycle, updates the damage estimates, and includes results on the Paris Accord and temperature-limited scenarios.

## 1. Background

### 1.1. Integrated Assessment Models.

Many areas of the natural and social sciences involve complex systems that link together multiple physical or social networks. This is particularly true for environmental problems such as climate change, which are intrinsically ones having firm roots in the natural sciences and requiring social and policy sciences to solve in an effective and efficient manner. As understanding progresses across the different fronts, it is increasingly necessary to link together the different areas to develop effective understanding and efficient policies. In this area, integrated assessment analysis and models play a key role. Integrated assessment models (IAMs) can be defined as approaches that integrate knowledge from multiple domains into an internally consistent framework. Their strength is that they can be used to estimate the social cost of carbon emissions (SCC), to solve for economically efficient policies, and to evaluate the costs and benefits of alternative scenarios.

### 1.2. DICE-2023.

This study introduces DICE-2023, an updated version of the DICE model (Dynamic Integrated model of Climate and the Economy) which has been widely used to estimate the SCC and evaluate climate policies across the literature and policy realm (1). The latest prior version was the DICE-2016 model (2–4).

DICE is based on a standard neoclassical model of optimal economic growth known as the Ramsey model. The DICE model augments the Ramsey model to include climate investments. In this augmented approach, society can give up consumption today to mitigate climate change and thus increase well-being in the future through avoided climate damages. The model contains all elements from economic activity and emissions through climate change to damages and policy in a manner that represents simplified best practice in each area.

The DICE model and its regional version, RICE (Regional Integrated model of Climate and the Economy), have gone through several revisions since their first development around 1990. The latest published versions are the RICE-2010 and DICE-2016 models. This study describes the revision of DICE, whereas an updated version of RICE-2022 has been developed by Zili Yang (5) 2022. We begin with a description of the DICE-2023 model, after which we provide the detailed equations. For details of the modeling technique and earlier approaches, see refs. 2–7.

## 2. Objectives of IAMs

IAMs can be divided into two general classes—policy optimization and policy evaluation models. The DICE model is primarily designed for policy optimization, although it can also be run as an evaluation model for given policies. In both settings, the approach is to maximize an economic objective function (the goal implicit in the problem). For the DICE model, the objective function refers to the economic well-being (or utility) associated with a path of consumption. Consumption is affected in the usual way by productivity and saving, but in the context of climate change, mitigation, adaptation, and damages will be the major factors impacting the differences in consumption over time.

Further, the world or individual regions are assumed to have well-defined preferences, represented by a social welfare function, which ranks different paths of consumption with associated mitigation and damages. The social welfare function is increasing in the discounted sum of the population-weighted utility of per capita consumption of each generation, with diminishing marginal utility of consumption. The level of per capita utility in each period is weighted by population and a discount factor.

The relative importance of different generations is affected by two central normative parameters: the pure rate of social time preference (generational discounting), and the elasticity of the marginal utility of consumption (the consumption elasticity for short). These parameters interact to determine the discount rate on goods, dimensionally the same as a real interest rate, which is critical for intertemporal economic choices.

An important feature of the 2023 modeling is to include the relative riskiness of climate investments such as abatement. Using the concept of climate beta (8), we adjust the discount rate on climate investments to reflect the presumption that they may have risk properties different from conventional investments.

## 3. Equations of the DICE-2023 Model

We next describe the equations of the model. We omit minor equations such as accounting identities.

### 3.1. Objectives.

To begin with, we assume that policies are chosen to maximize a general concept of economic welfare. More precisely, we maximize a social welfare function, *W,* which is the discounted sum of the population-weighted utility of per capita consumption.

Eq. **1** shows the objective function, which is standard in modern theories of optimal economic growth.

[1] $$\begin{array}{c} W=\sum_{t=2020}^{T\text{max}}U\left[c\left(t\right)\right]L\left(t\right)\Pi \left(t\right)\\ \;=\sum_{t=2020}^{T\text{max}}[c\left(t{)}^{1-\phi }/\left(1-\phi \right)\right]L\left(t\right)\Pi \left(t\right). \end{array}$$

In Eq. **1**, *W* is the welfare total, *U* is the utility function, *c*(*t*) is per capita consumption per period *t* = 2020, 2025, 2030…, *L* (*t*) is population and labor inputs, and Π(t) is the discount factor.

Each period’s preferences are represented by the utility of consumption, with a constant elasticity of the marginal utility of consumption, ϕ, which represents the extent of substitutability of the consumption of different years or generations. Additionally, the elasticity is distinct from personal behavioral characteristics and is not used in the DICE model to represent risk aversion.

In the updated DICE specification, the discount factor $\Pi \left(t\right)$ contains three elements: (i) a pure rate of social time preference, *ρ,* which reflects the welfare weights on the utilities of different generations, (ii) a precautionary term reflecting consumption growth uncertainty, and (iii) an adjustment for the nondiversifiable risk of climate investments. Elements (ii) and (iii) are introduced in DICE-2023 and are corrections to reflect uncertainty through the use of certainty-equivalent discount rates. This term is used to designate the single discount rate delivering the same discount factor as the expected value from the distribution of uncertain future discount rates.

The precautionary effect in (ii) is associated with the uncertainty about the trend growth of per capita consumption. Based on different studies, we assume the average growth rate of per capita consumption from *t* = 0 to *t* = *T* is normally distributed with a SD $\left(\sigma_{C}\right)$ of 1%-point/year. The adjustment in (iii) for risky climate investments is based on the concept of the climate beta, $\beta^{\text{CLIM}}$ (8). The climate beta measures the extent to which climate investments (such as renewable power) share the nondiversifiable risk characteristics of economy-wide investments. When $\beta^{\text{CLIM}}=0,$ the risks on climate investments are uncorrelated with market returns; if $\beta^{\text{CLIM}}=1,$ climate investments have risk properties of the aggregate economy. Based on our review, we assume that $\beta^{\text{CLIM}}=0.5,$ which implies an intermediate correlation with market risks. We further assume, based on historical data, a near-term risk-free real rate of return of 2%/year and the economy-wide nondiversifiable risk premium of π = 5%/year.

Using these assumptions, we substitute a time-varying risk-adjusted time preference parameter, $\rho^{*}\left(t\right)$, for *ρ*, where $\rho^{*}\left(t\right)$ = $\rho -½\;\phi^{2}{\sigma_{C}}^{2}t+\beta^{\text{CLIM}}\pi .$ When calibrated to the parameters noted above, this approach yields a near-term real rate of return of 4.5%/year, declining over time. A full discussion of the approach is contained in *SI Appendix*, *Appendix C* and the Background Note on Rates of Returns and Discounting.

### 3.2. Population, Output, and Productivity.

The DICE model is a standard one-sector model with output (Q) determined by a Cobb-Douglas production function in capital and labor (K and L) with growing total factor productivity (A). Output is reduced by abatement and damages as shown in Eq. **2**, where Ω and Λ represent climate damages and abatement costs, respectively, and are discussed in the next section.

[2] $$Q\left(t\right)=\left[1-\Lambda \left(t\right)\right]\left[1-\Omega \left(t\right)\right]A\left(t\right)K{\left(t\right)}^{\gamma }L{\left(t\right)}^{\left(1-\gamma \right)}.$$

Population and the labor force are exogenous and are based on UN projections. Output is measured in PPP exchange rates using World Bank and IMF estimates. Future productivity growth is based on estimates from refs. 9–12, see *SI Appendix*, *Appendix J* for details. Technological change is exogenous and takes two major forms: economy-wide technological change and carbon-saving technological change. Carbon-saving technological change is represented in two ways: first, as reducing the baseline ratio of CO_2_ emissions to output and, second, as reducing the cost of the backstop technology.

### 3.3. Damages.

Eq. **3** represents the economic impacts or damages from climate change, which has been one of the thorniest issue in climate-change economics. Providing reliable estimates of the damages from climate change over the long run has proven extremely difficult, and we examine alternative approaches.

[3] $$\Omega \left(t\right)\;=\psi_{1}T_{\mathrm{AT}}\left(t\right)+\psi_{2}[T_{\mathrm{AT}}\left(t\right){]}^{2}.$$

The damage function is a quadratic function of global temperature increase since preindustrial times (1765), $T_{\mathrm{AT}}\left(t\right)$. It is based on three key assumptions: i) The increase in global mean surface temperature from preindustrial levels is assumed to be a reasonable sufficient statistic for damages. This specification omits or captures only indirectly cumulative effects (such as the effects of prolonged rather than instantaneous warming on sea-level rise) and also omits effects that depend on the speed of temperature change. ii) Damages scale proportionately with global output. iii) Damages are quadratic in warming, in line with recent reviews (13, 14) but with potential limitations discussed below. The estimates are based on three components.

The first component is an updated literature synthesis as described in *SI Appendix*, *Appendix F*. DICE-2023 builds on (13) and adds studies published since that review. The update is based on a survey by ref. 15, which overlaps closely also with global damage studies reviewed by the IPCC’s AR6 (16). The updated results imply a 1.6% GDP-equivalent loss at 3 °C warming over preindustrial temperatures, up from 1.2% in the review for DICE-2016. It is important to note that surveyed studies generally omit many climate change impact channels, such as biodiversity loss, ocean acidification, extreme events, and social unrest.

The second component, based on a comprehensive study of tipping points (17), adds a 1% output loss at a 3 °C warming. The third component is a judgmental adjustment for excluded impacts totaling 0.5% output loss at 3 °C warming. This adjustment reflects concerns over missing sectors, climate change impacts not yet reliably quantified in the literature, uncertainty, and recent research that is not reflected in our synthesis of aggregate damage estimates.

Each of the three components is assumed to be proportional to output and quadratic in the temperature change from preindustrial times. In total, damages are estimated to be 3.1% of output at 3 °C warming and 7.0% of output at 4.5 °C warming. The resulting damage coefficient is almost twice as large as in DICE-2016, resulting in more stringent emissions reductions and a large increase in the social cost of carbon.

Note that the damage function has been calibrated for damage estimates with temperature increases up to 4 °C and is not well-suited for temperature increases above that range. The evidence is very limited for warming beyond 4 °C, and the quadratic functional form in Eq. **3** does not reflect potential concerns about threshold damages.

### 3.4. Abatement.

The abatement cost equation in Eq. **4** is a reduced-form type model in which the ratio of the costs of emissions reductions to output, Λ(t), is a polynomial function of the emissions-control rate, μ(*t*).

[4] $$\Lambda \left(t\right)=\theta_{1}\left(t\right)\mu {\left(t\right)}^{\theta_{2}}.$$

The intercept, $\theta_{1}\left(t\right)$, represents the fraction of output that is required to reduce emissions to zero.

The DICE model includes a backstop technology, which is a set of technologies that can replace all fossil fuels, albeit at a relatively high price. These technologies might be solar or wind power, safe nuclear power, or some as-yet-undiscovered source. Conceptually, at the cost of the backstop technology, the economy achieves zero net carbon emissions.

Two revisions in the current version are noteworthy. Estimates of the cost of the backstop technology are controversial, with the DICE model having a high backstop cost relative to some estimates of the cost of renewables or carbon capture. The cost function is derived from highly detailed process models. Examining estimates of the marginal cost of scenarios with zero net emissions, we can estimate the marginal cost of the backstop technology. A statistical analysis from the results of the ENGAGE study (18, 19) indicates a median backstop price of $515/tCO_2_ in 2019$ in 2050, which is the earliest year that most models can reach zero net emissions. Models assume improvements over time in the technologies needed to attain zero emissions. The decline rate of the cost of the backstop technology is assumed to be 1%/year from 2020 to 2050, and then 0.1%/year after that.

The backstop technology is introduced into the model by setting the time path of the parameters in the abatement-cost Eq. **4** so that the marginal cost of abatement at a control rate of 100 percent is equal to the backstop price. By construction, the cost of a zero-emissions policy is determined by the cost of the backstop technology and the emissions-output ratio. With the assumed parameters, the cost of net-zero emissions is 11% of output in 2020, declining at 1.7% per year from 2020 to 2100 to 2.7% of output in 2100.

The other revision is the inclusion of emissions other than industrial CO_2_. This addition is basically a scalar increase in the abatement cost function. For further discussion, see the section on emissions below as well as *SI Appendix*, *Appendix E*.

### 3.5. Emissions.

DICE-2023 has a major revision in its treatment of greenhouse gas (GHG) emissions. In earlier versions, only industrial CO_2_ emissions were controllable (abatable), while other GHGs and forcings were taken to be exogenous. The current version includes all abatable emissions in the endogenous category and excludes only a small fraction of forcings as nonabatable emissions. *SI Appendix*, *Appendix G* contains a full discussion of the methods.

The lion’s share of GHG emissions is from CO_2_. However, a large suite of processes and gases also contribute to radiative forcings. According to IPCC AR6, total CO_2_-equivalent (CO_2_-e) abatable emissions are 140% of industrial emissions in 2020, declining to 121% of industrial CO_2_ emissions in 2100. This ratio indicates the increase in abatable emissions in DICE-2023 compared to DICE-2016. The cost function is drawn from studies of the abatement cost function for non-CO_2_ emissions. This extension allows a larger potential abatement and the possibility of attaining more ambitious targets.

Projections of baseline emissions are a function of total output, time, a time-varying emissions-output ratio, and the emissions-control rate. The baseline emissions control rate reflects current policy, which we estimate to be about 5%, or a carbon price of about $6/tCO_2_. There is no major change in the function form of the abatement-cost function from earlier DICE models, but the extension to nonindustrial CO_2_ emissions is an addition to the current vintage and is based on studies of the abatement-cost function of nonindustrial CO_2_ and abatable non-CO_2_ GHGs.

The final two equations in the economic block are the emissions equation for CO_2_ and that for abatable non-CO_2_ GHGs:

[5] $${\text{ECO}}_{2}\left(t\right)=\left[\sigma \left(t\right)Y\left(t\right)+{\text{ECO}}_{2Land}\left(t\right)\right]\;\left[1-\mu \left(t\right)\right],$$

[6] $$\begin{array}{c} {{\text{ECO}}_{2}e}_{{\text{NonCO}}_{2}\text{GHGabate}}\left(t\right)\;\\ =\left[{\text{ECO}}_{2}e_{{\text{NonCO}}_{2}{\text{GHGabate}}_{\text{base}}}\left(t\right)\right]\;\left[1-\mu \left(t\right)\right]. \end{array}$$

Eq. **5** defines total CO_2_ emissions per period. The first term is industrial emissions, given by the level of no-controls carbon intensity, *σ*(*t*), times output. *σ*(*t*) is taken to be exogenous and declines initially at a rate of 1.5% per year. The second term is land-use emissions of CO_2_, which decline by 2% per year. Actual CO_2_ emissions are base emissions times (one minus the emissions-control rate) or [1−μ(*t*)]. Eq. **6** represents abatable non-CO_2_ GHG emissions measured on a CO_2_-equivalent (CO_2_-e) basis. These emissions equal uncontrolled emissions (based on the SSP2 scenario (20)) times [1−μ(*t*)]. Our treatment assumes the same control rate on CO_2_ and non-CO_2_ abatable emissions. Total abatable emissions in CO_2_-e units are given by the sum of (**5**) and (**6**).

### 3.6. Geophysical Sectors.

A key feature of IAMs is the inclusion of geophysical relationships that link the economy with the different forces affecting climate change. In the DICE model, these relationships include the carbon cycle, a radiative forcing equation, and the climate-change equations. The purpose of including these is that they operate in an integrated fashion rather than taking inputs as exogenous from other models or assumptions.

This block of equations links economic activity and greenhouse gas emissions to the carbon cycle, radiative forcings, and climate change. As with the economics, the modeling philosophy for the geophysical relationships has been to use parsimonious specifications so that the theoretical model is transparent and so that the optimization model is empirically and computationally tractable and robust.

For purposes of the carbon/forcings/climate modules, CO_2_ emissions are linked to the carbon cycle and thence to forcings. The other GHGs are linked directly to forcings and short-circuit the atmospheric chemistry.

### 3.7. Carbon Cycle.

The carbon cycle and climate model are key components of any IAM. DICE-2023 has made a major change in the treatment of these modules, particularly the carbon cycle. Earlier versions of DICE and most other IAMs used linear carbon-cycle structures. While these approaches seemed acceptable as a simplification, they did not allow for the important finding that the ability of nonatmospheric sinks to absorb CO_2_ declines with higher emissions (21, 22). The latest and most extensive multimodel carbon-cycle comparison by ref. 23 showed that the atmospheric retention at 100 y would be 70% for a pulse of 5,000 billion tons of carbon (GtC) compared to only 30% for a pulse of 100 GtC.

The major structural revision of DICE-2023 is the introduction of the DFAIR module, the DICE version of the FAIR or Finite Amplitude Impulse-Response model developed by Millar et al. (24), which represents the dynamics of the carbon cycle. The FAIR model is based on a linear four-reservoir impulse-response model of the response of CO_2_ concentrations to emissions. A key innovation is the structural parameter α(*t*), which increases the fraction of total CO_2_ emissions that resides in the atmosphere as cumulative CO_2_ emissions increase. While the reservoirs may have geophysical names (permanent, long, etc.), they have no physical or structural interpretation but are variables in reduced-form dynamic equations and may take negative values.

Simulations reported in *SI Appendix*, *Appendix D* indicate that the DFAIR model tracks the historical emissions-concentrations paths closely, as well as small emissions pulses. However, the DFAIR atmospheric retention for very large pulses (e.g., the 5,000 GtC pulse in ref. 23) tracks the full carbon-cycle models poorly.

The DFAIR equations are the following. Eq. **7** is the set of equations for the four reservoirs, whose contents are $R{\;}^{i}\left(t\right)$. We note that only CO_2_ emissions (industrial and land-based) enter the carbon cycle, that is, CO_2_-equivalent emissions from other gases are not included in the emissions term *E(t)*. Eq. **8** then sums the four reservoirs to obtain atmospheric CO_2_, MAT(t). Eq. **9** provides the equation for accumulated CO_2_ in nonatmospheric sinks, defined as Cacc(t). Eq. **10** yields the predicted 100-y integrated impulse response function IRF100(t) and (11) implicitly defines the saturation parameter α(t). All equations are straightforward to calculate except for (11).

[7] $$\Delta R^{i}\left(t+1\right)=\xi_{i}E\left(t\right)-\left[\frac{R^{i}\left(t\right)}{\alpha \left(t\right)\tau_{i}}\right],i=1,...,4,$$

[8] $$MAT\left(t\right)-MAT\left(1765\right)=\sum_{i=1}^{4}R^{i}\left(t\right),$$

[9] $$Cacc\left(t\right)=\sum_{v=1765}^{t}E(v)-\left[MAT\left(t\right)-MAT\left(1765\right)\right],$$

[10] $$\text{iIRF}100\left(t\right)=\varsigma_{0}+\varsigma_{C}Cacc\left(t\right)+\varsigma_{T}T_{\mathrm{AT}}\left(t\right),$$

[11] $$\text{iIRF}100\left(t\right)=\sum_{i=1}^{4}\alpha \left(t\right)\xi_{i}\tau_{i}\{1-\exp[-100/(\alpha \left(t\right)\tau_{i})]\}.$$

The variables are *MAT* = atmospheric concentrations, *R^i^ =* carbon content of reservoir *i*, *E* = emissions of CO_2_, iIRF100 = 100-y integrated impulse response value, *Cacc* = accumulated carbon stock in the land and ocean, *α* = scaling factor for carbon reservoirs, ξ_i_ = fraction of emissions entering reservoir *i*, and τ_i_ = time constant for reservoir *i*. Note that values of *R, Cacc*, and *E* are all zero in 1765. The values of the parameters are described in *SI Appendix*, *Appendix D* and the Background Note on DFAIR.

### 3.8. Climate Equations.

The other equations of the climate system contain the relationships for radiative forcing and for global mean temperature. These specifications are similar to earlier versions of the DICE model but update the parameters and change the structure to parallel the treatment in ref. 24.

DICE employs a small structural model that captures the basic relationship between GHG concentrations, radiative forcing, and the dynamics of climate change. Accumulations of GHGs lead to warming at the earth’s surface through increases in radiative forcing. The relationship between GHG accumulations and increased radiative forcing is derived from empirical measurements and climate models, as shown in Eq. **12**.

[12] $$\begin{array}{c} F\left(t\right)=F_{{\text{CO}}_{2}2x}\left\{{\text{log}}_{2}\left[MAT\left(t\right)/MAT\left(1765\right)\right]\right\}\\ \;+F_{\mathrm{ABATE}}\left(t\right)+F_{\mathrm{EX}}\left(t\right). \end{array}$$

*F*(*t*) is the change in total radiative forcings of GHGs since 1765 from anthropogenic sources such as CO_2_ and other GHGs. *F*_EX_(*t*) is exogenous forcings from nonabatable GHGs and other sources, and *F_ABATE_(t)* is the forcings resulting from abatable non-CO_2_ GHGs (see Eq. **6** and *SI Appendix*, *Appendix G*). The equation uses estimated carbon stocks in the year 1765 as the preindustrial equilibrium.

The climate module in Eqs. **13** through (**15**) uses a two-box model of the temperature response to radiative forcing developed by IPCC AR5 and parameterized in ref. 24. DICE-2023 further adjusts the parameters to match the equilibrium climate sensitivity (ECS) and transient climate response (TCR) to the centers of the IPCC Sixth Assessment Report (25) ranges of 3.0 °C for ECS and 1.8 °C for TCR, respectively. The equations are:

[13] $$\begin{array}{c} Tbox1\;(t+1)=Tbox1(t)\;\exp(-5/d1\;)+teq1\;F\;(t+1)\;\\ \;[1-\exp\;(-5/d1\;)], \end{array}$$

[14] $$\begin{array}{c} Tbox2\;(t+1)=Tbox2(t)\;\exp(-5/d2\;)\\ +teq2\;F\;(t+1)\;[1-\exp(-5/d2\;)], \end{array}$$

[15] $$T_{\mathrm{AT}}\left(t\right)=Tbox1\;\left(t\right)+Tbox2\;\left(t\right).$$

The increase in global mean surface temperature, *T_AT_(t)*, is computed as the sum of two components, *Tbox1* and *Tbox2.* These are the contributions to temperature increase due to processes of the deep and upper ocean, respectively. The FAIR model assumes a neutral biosphere and is therefore likely to overestimate atmospheric accumulation in the early years. The parameters *d1* and *d2* are time lags for the two temperature boxes (in years). The parameters *teq1* and *teq2* are the diffusion rates for the boxes (in m^2^K/W). Note that the equilibrium climate sensitivity is given by $ECS=F_{CO22x}\left(teq1+teq2\right)=3.93\times \left(0.324+0.440\right)=3.0$. The model’s transient climate response is 1.80 °C (for the complex formula defining the value, see ref. 24, Eq. **5**).

This completes the description of the DICE-2023 model. A full discussion of the DFAIR module—including updates such as initial conditions relevant for 2020—is in *SI Appendix*, *Appendix D*. Computational and algorithmic aspects, with comparisons to earlier versions, are discussed in *SI Appendix*, *Appendix K*.

## 4. Scenarios to Evaluate

Integrated assessment models such as DICE have a wide variety of applications. Among the most important ones are the following: 1) making consistent projections, i.e., ones that have consistent inputs and outputs of the different components of the system; 2) calculating the impacts of alternative assumptions on important variables such as output, emissions, temperature change, impacts, prices, and economic growth; 3) tracing through the effects of alternative policies on all variables in a consistent manner; 4) estimating the costs and benefits of alternative strategies, and 5) estimating the uncertainties associated with alternative variables and strategies. The current study presents a suite of scenarios as follows.

### 4.1. Baseline.

This scenario contains estimates of current climate policies, and the trends of current policies as of 2023 are extended indefinitely. This approach is standard for forecasting, say of government budgets, and is appropriate for a world of evolving climate policies. The baseline assumption is that the global average carbon price on CO_2_ emissions is $6/tCO_2_, growing at 2.5% per year.

### 4.2. No Controls.

We sometimes will refer to a no-controls path. This is a scenario with a carbon price equal to $0. It is for reference in calculating variables and is not used as a scenario for evaluation.

### 4.3. Cost–Benefit Optimal (C/B Optimal).

In this scenario, climate change policies maximize economic welfare according to the principles of cost–benefit analysis, with full participation by all nations starting in 2025. The C/B optimal scenario involves a balancing of the present values of the costs of abatement and the benefits of reduced climate damages. Although the underlying assumptions are highly optimistic, this scenario provides an efficiency benchmark against which other policies can be measured. (Note that this scenario was called optimal in earlier versions. The term cost–benefit was added to emphasize that it relies on monetized impacts and uses standard economic approaches to welfare maximization.)

### 4.4. Temperature-Limited.

In this scenario, the C/B optimal policies are undertaken subject to a further (precautionary) constraint that global temperature does not exceed 2 °C (or other targets) above preindustrial levels. The temperature-limited scenarios are variants of the C/B optimal scenario that build in a precautionary temperature constraint.

### 4.5. Alternative Discount Rates.

The assumptions about discounting are highly controversial and have major implications for the SCC and for policies. We consider alternatives to the standard approach discussed above by setting constant discount rates of 1%, 2%, 3%, 4%, or 5% per year (see *SI Appendix*, *Appendix C.3* for details).

### 4.6. Alternative Damage Function.

This scenario uses an alternative damage function quantification based on Howard and Sterner (26, 27). The damage function has the same structure as the DICE version. While there are several potential results to choose from in Howard and Sterner, a reasonable middle ground of their preferred estimates is a 9% damage/output ratio at a 3 °C increase. This temperature-damage coefficient is 3 times larger than the one used in the current DICE model.

### 4.7. Paris Accord Extended.

The Paris Accord of 2015 codified a policy that would aim to limit climate change to 2 °C above preindustrial levels. To achieve this goal, countries agreed to make their best efforts through nationally determined contributions. This scenario assumes that countries meet their objectives in 2030 according to their revised pledges as of summer 2022 and projects slightly less than ½ percentage point increase per year in the control rate from 2030 to 2100. This scenario further assumes that pledges are implemented through internationally harmonized carbon prices. It should be emphasized that any projections beyond 2030 are not based on country commitments and are therefore conjectural.

### 4.8. Major Constraints.

All scenarios have some important implementation constraints built in. One constraint is that climate policies have limits on implementation. These involve emission control rates increasing at a maximum of 12 percentage points per 5-y period. Additionally, the emissions control rate is limited to 100% through 2120 and to 110% after that. The control limits are drawn from runs that stress high-resolution IAMs with extremely high carbon prices. Finally, all scenarios assume 100% country participation with harmonized and comprehensive carbon prices. These assumptions about policy, particularly participation and harmonization, are highly optimistic and will lead to lower costs and better implementation of targets than scenarios where country actions and international agreements fall short of the ideal. Note as well that we do not consider solar-management geoengineering, which raises a host of other issues.

## 5. Results

We now report on a set of representative results. All scenarios ran smoothly with the exception of the 1.5 °C limit, which is infeasible within the constraints of realism and of the technologies considered (such as the omission of geoengineering). To meet the 1.5 °C target, emissions would be required to fall virtually to zero in the next 5 y. This would entail either a deep depression (output declining by around 75%) or an implausibly sharp increase in emissions reductions (by at least 50% within a decade). The scenario is so far from what any economic model can hope to capture realistically that it is best thought of as infeasible.

### 5.1. Emissions, Concentrations, and Temperature.

**Fig. 1.**
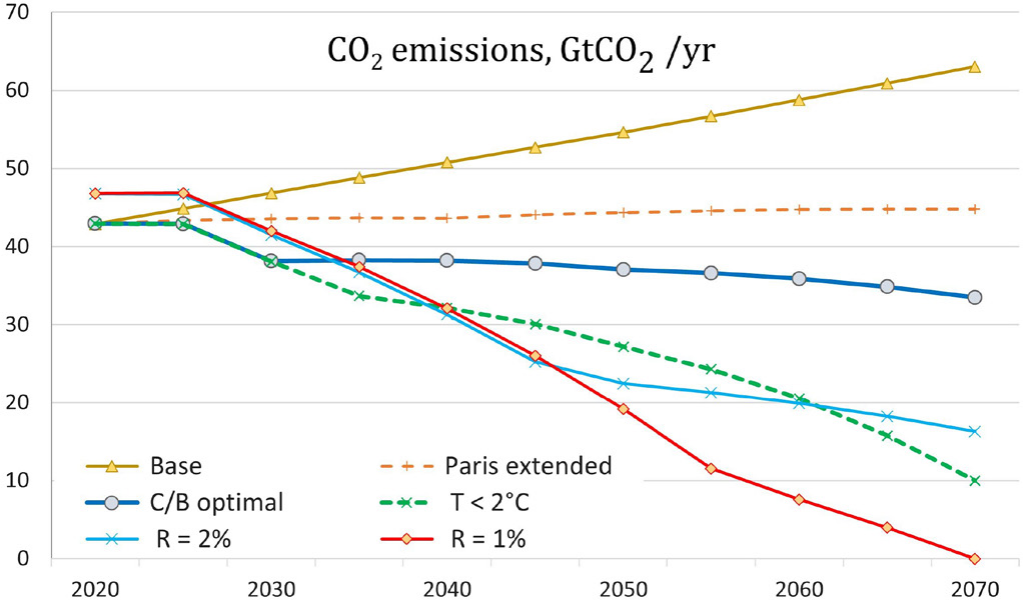
Results for CO_2_ emissions in different scenarios. Note that emissions in low-discount scenarios are higher in early years because of higher output due to higher savings rates. In Figs. 1–3, the label “R = X%” is the scenario with a constant discount rate of X% per year.

For the major results, we focus on the baseline, C/B optimal, 2 °C, and Paris policies. Fig. 1 and *SI Appendix*, *Appendix* Table A-1 report the results for CO_2_ emissions under different scenarios. The current policy baseline implies increasing emissions in the coming decades, in stark contrast with the declining emissions needed to achieve any of the policy objectives.

**Fig. 2.**
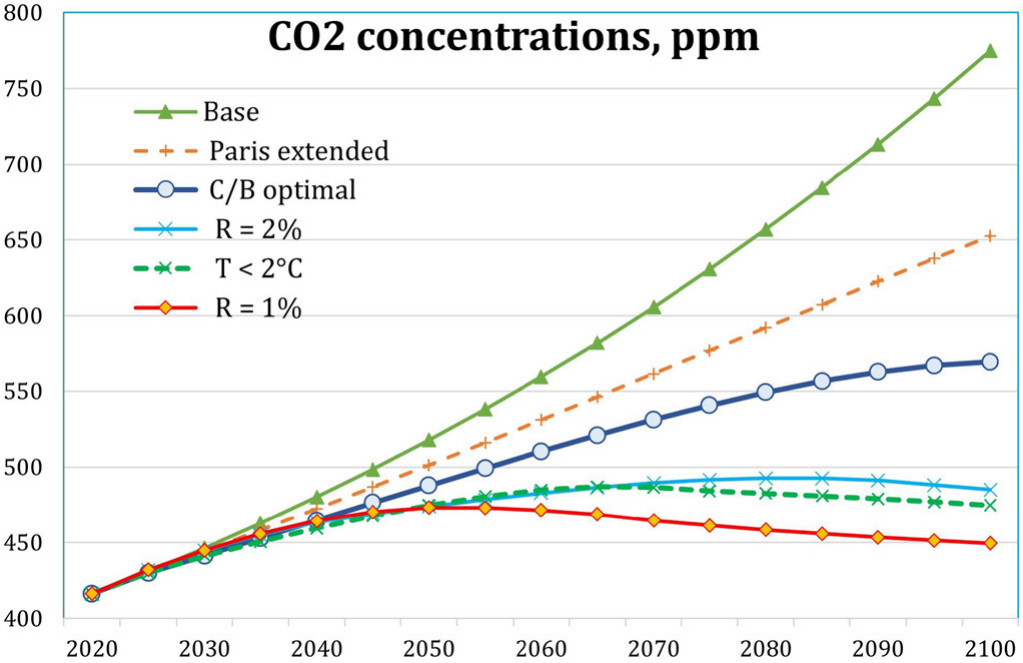
CO_2_ concentrations in different scenarios.

The Fig. 2 and *SI Appendix*, *Appendix* Table A-2 report the results for CO_2_ concentrations for different scenarios. The 2 °C target will require stabilization of CO_2_ concentrations at slightly more than 10% above current levels. Note that the Paris Accord will reduce concentrations about one-third of the way to the 2 °C target.

**Fig. 3.**
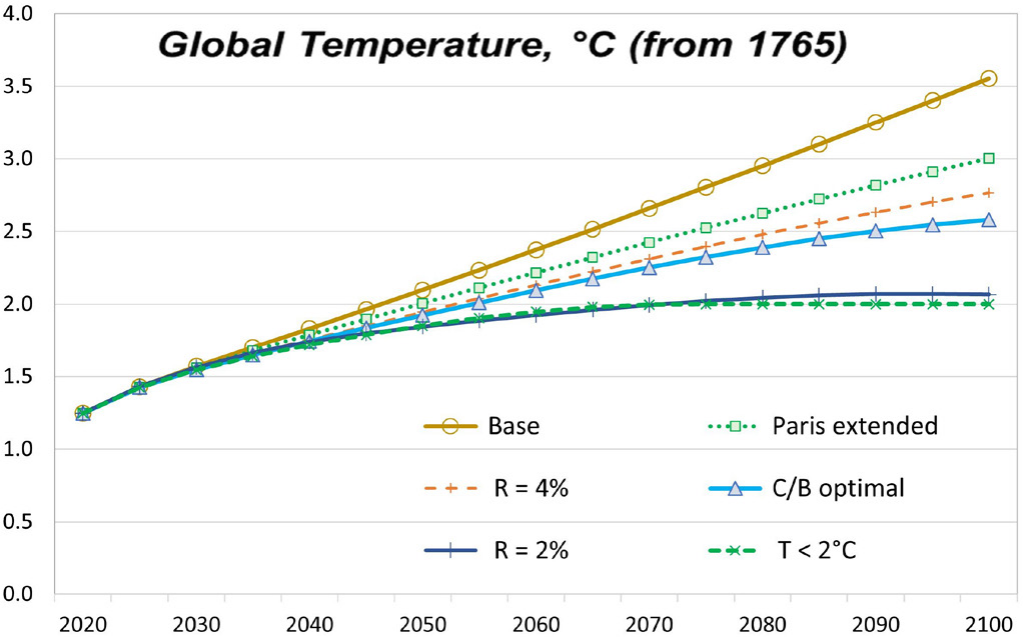
Global temperature increases in different scenarios.

The Fig. 3 and *SI Appendix*, *Appendix* Table A-3 report the results for the increases in global temperature in different scenarios. The temperature change for 2100 in the baseline (current policy) run is 3.6 °C. The 2100 temperature change for the C/B optimal run is 2.6 °C. The C/B optimal temperature change is significantly above 2 °C run because damages do not have a kink at the threshold 2 °C temperature change, but the C/B optimal temperature path would depend as well on other key parameters such as abatement costs. Among the broader scenarios considered, the 2 °C target does pass the cost–benefit test in cases with sufficiently low discounting (2%) or the alternate damage function. In 2100, the Paris Accord reduces the temperature increase by one-third of the way from the base path to the 2 °C target. Note that these temperature increases are slightly above conventional measures because they use the preindustrial (1765) baseline rather than later benchmarks.

### 5.2. Policies and Impacts on Income.

We next discuss key policy variables. Table 1 shows results for the emissions control rate across the main scenarios (with further results reported in *SI Appendix*, *Appendix* Table A-4 and *Appendix* Fig. A-1). Recall that this applies to all of the CO_2_ emissions as well as abatable non-CO_2_ GHGs. The emissions control rates across all scenarios start at 5% in 2020. In the base case, they remain low because of the weak level of current policy. The emissions control rates for policies in 2050 are 27%, 39%, and 55% for the Paris, C/B optimal, and 2 °C targets; and in 2100 are 57%, 84%, and 99% for the Paris, C/B optimal, and 2 °C targets. These necessary control rates are low relative to other estimates because of the comprehensive nature of the controlled gases and because the runs assume complete efficiency and participation.

**Table 1.** Emissions control rates (percent of both CO_2_ and abatable non-CO_2_ emissions avoided) and total global wealth (present value of consumption, 2019 US$) across policy scenarios

Scenario	Emissions control rate			Present value of consumption	Difference from base
	2020	2050	2100	(Trillions of 2019 US international $)	
Base	5%	10%	22%	6,540	0.0
C/B optimal	5%	39%	84%	6,659	119.7
T < 2 °C	5%	55%	99%	6,647	107.3
Paris, updated 2022	5%	27%	57%	6,625	85.4

“Wealth” is the present value of global consumption of goods and services. They are benchmarked so that the present value of consumption in that scenario is the value of the objective function in the baseline scenario.

The carbon prices associated with these emissions control rates start at an estimated baseline price of $6/tCO_2_ (2019$) for 2022. These reflect either the trading price for universal capped emissions or the harmonized level of universal carbon taxes. In order to implement the C/B optimal, Paris, and 2 °C emissions reductions targets, global carbon prices must rise to $115/tCO_2_, $63/tCO_2_, and $200/tCO_2_ by 2050, respectively (see *SI Appendix*, *Appendix* Fig. A-2 for further results on carbon prices). In these calculations, the average carbon prices are modest relative to other estimates primarily because the emissions control rates are lower.

In the baseline (current policy) scenario, annual losses reach 4.4% of output by 2100. The extended Paris program improves on the baseline, with losses of 3.1% of output in 2100. The C/B optimal program reduces damages by half compared to the current policy scenario, with a damage-output ratio of 2.3% in 2100. The 2 °C limit scenario has a damage-output ratio of 1.4%.

Table 1 shows the total wealth in each scenario. These are calculated as the present value of consumption (technically, this is the present value of utility calibrated to first-period consumption). The stakes in an efficient program are clearly substantial. The C/B optimal program increases wealth by $120 trillion, or slightly less than one year’s output. The 2 °C and Paris programs also make substantial improvements, increasing wealth by around $107 and 85 trillion in present value, respectively. The wealth estimates of the policies shown in Table 1 are relatively close because the emissions paths are similar and because the wealth hill is flat at the policy maximum.

### 5.3. The Social Cost of Carbon.

The most important single economic concept in the economics of climate change is the social cost of carbon (SCC). This term designates the discounted value of the change in consumption caused by an additional ton of carbon dioxide emissions or its equivalent. The SCC has become a central tool used in climate change policy, particularly in the determination of regulatory policies that involve greenhouse gas emissions. While estimates of the SCC are necessarily complex, IAMs are ideally suited to calculate them because of their comprehensive and internally consistent structure.

The definition of the SCC is the derivative of the objective function (or of the present value of consumption) with respect to CO_2_ emissions in a given year. In actual calculations, the estimates are calculated as the ratio of shadow prices (which are algorithmic derivatives) in the different scenarios.

Table 2 and *SI Appendix*, *Appendix* Fig. A-3 show estimates of the SCC from DICE-2023. The SCC in the baseline run is $66/tCO_2_ for the 2020 period (in 2019 international $). This is above the SCC for the C/B optimal run of $50/tCO_2_ because damages are smaller in the C/B optimum. It is far below the SCC for the 2 °C run of $76/tCO_2_. The higher SCC in the temperature-limited run reflects the economic interpretation that a tight temperature limit is equivalent to a damage function with a sharp kink at the temperature limit and therefore to a sharply higher damage function above 2 °C.

**Table 2.** Social cost of carbon, alternative scenarios (2019$/tCO_2_)

	Social cost of carbon ($/tCO_2_, 2019$)		
Scenario	2020	2025	2050
C/B optimal	50	59	125
T < 2 °C	75	89	213
T < 1.5 °C	3,557	4,185	16,552
Alt damage	124	146	281
Paris extended	61	72	159
Base	66	78	175
R = 5%	32	37	74
R = 4%	49	58	107
R = 3%	87	102	172
R = 2%	176	207	302
R = 1%	485	571	695

This table shows the importance of discounting and alternative damage estimates on the SCC. It includes the SCC for the 1.5 °C scenario to indicate the cost induced by the catastrophic loss of output to reach the target. The label “R = X%” is scenario with a constant discount rate of X% per year.

One of the most instructive findings involves the importance of discounting for the SCC and other policies. Table 2 shows alternative estimates of the SCC in the DICE-2023 scenarios and particularly emphasizes the powerful impacts of discounting and climate damages on the SCC.

**Fig. 4.**
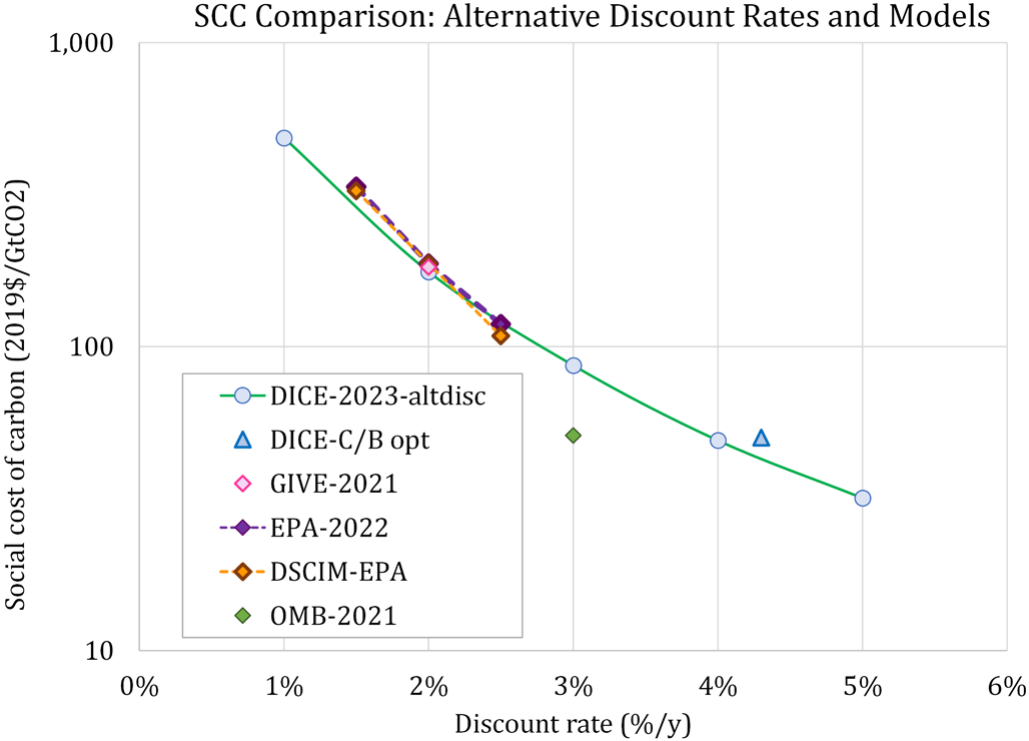
Social cost of carbon, 2020, alternative discount rates and models (2019$/tCO_2_). The figure shows the relationship between the discount rate on goods and the SCC in different scenarios of the DICE-2023 model and several other models. Results in order of the list are as follows: DICE 2023-altdisc is the solid green line connecting the runs for constant discount rates in DICE-2023; DICE- C/B-opt is the DICE-2023 estimate for the C/B optimal scenario along with the average discount rate for the period 2020 to 2050; GIVE-2021 is the estimate from the GIVE model (28); EPA-2022 are the draft EPA social costs of greenhouse gas estimates based on an overall assessment (29); DSCIM-EPA are the estimates specific to a damage module based on the DSCIM framework (30); and OMB-2021 (31) is a preliminary OMB estimate based on earlier methods which did not reflect the changes introduced in 2022. Discount rates for EPA values correspond to near-term rates in their assessment.

Additionally, Fig. 4 compares DICE estimates of the year-2020 SCC with several other current values, as explained in the legend. The surprising conclusion from Fig. 4 is that the estimates from different sources are quite close conditional on the discount rate. Fig. 4 highlights the importance of the discount rate in determining the SCC.

## 6. Modeling Issues in DICE-2023

This discussion makes it clear that DICE-2023 is a highly simplified representation of the complex economic and geophysical realities. While small and comprehensive models have many advantages, they also have major shortcomings because of their simplifications. We discuss several issues in this final section.

One important set of issues concerns taxation (32). The simplest IAMs ignore the structure of the tax system. This is particularly important for energy and capital taxes and subsidies, which have large effects on energy use and on the rates of return used in making long-term decisions in the energy sector. These distortions may have major implications for efficient intertemporal pricing of consumption, the discount rate, and the SCC. One vexing issue is the appropriate tax rate for long-term discounting when consumers face both taxes on capital income and liquidity constraints. The complexity of tax structures is just one of many inefficiencies and externalities in the real-world economy that are not reflected in the DICE model.

Additionally, many simplifications are also buried in the functional forms of models. One simplification is the use of a single commodity to represent all commodities in all sectors, which is particularly restrictive in the context of international trade, where the essence of trade is the heterogeneity of goods across regions. Additionally, the assumed Cobb-Douglas function overestimates substitution in some areas and underestimates it in others. This function allows a degree of smoothness in substitution that is not present when there are only a small number of processes, in which case an activity analysis framework would be preferable.

Finally, we note specific concerns. We note that the carbon-cycle component of the DFAIR model included here is a recent development and has not been widely used or compared with alternatives. Additionally, the damage module remains highly uncertain as climate impact estimation techniques diverge across studies and syntheses. A further concern is the assumption of perfect implementation of climate policies, which are assumed to be universal and harmonized across and within all countries. This assumption will lead to policies that are unrealistically effective since some countries are unlikely to join, and policies within and across countries are almost certain to diverge. Also, while the introduction of investment risk using the climate beta is an innovative approach that has important implications for the appropriate discount rate, this approach depends critically on empirical estimates of the climate beta, which have a thin empirical basis and have difficulties in accounting for the equity-premium puzzle. Similarly, the discount rate adjustment for growth uncertainty is an addition to DICE-2023, but its quantification depends on the specification of growth uncertainty and introduces the potential for time inconsistency in the planner’s problem. We take this to be a learning effect but note that the simplified representation of these uncertainties in a deterministic model cannot capture reality fully.

We must put these concerns about oversimplification in the context of the questions that are being asked. The purpose of models is not to be an exact replica of real-world processes. Aside from the impossibility of achieving that goal, greater detail is often less valuable for many purposes. For example, if we are concerned about the long-run intertemporal tradeoffs between consumption today and consumption in the future, a simple model can illustrate the issues cleanly.

## 7. Summary of Key Policy Conclusions

We conclude with three results relevant for climate policy. First, both current policies (base run) and the extended Paris Accord fall short of limiting global warming to 2 °C or to the cost–benefit optimal level. Second, the economic stakes in global climate policy are substantial, with estimated net present value of economic benefits around $120 trillion from the cost–benefit optimal policy. Third, once differences in discounting are considered, the baseline DICE-2023 estimate of the social cost of carbon ($66/tCO_2_ for 2020) aligns closely with other recent estimates (28–30).

## Supplementary Material

Appendix 01 (PDF)

## Data Availability

All study data are included in the article, and/or publicly available (33).
