# Supplementary material for: Policies, projections, and the social cost of carbon: Results from the DICE-2023 model
Source: Proc Natl Acad Sci U S A. 2024 Mar 19;121(13):e2312030121. doi: 10.1073/pnas.2312030121 (PMC10990119; doi:10.1073/pnas.2312030121)
Supplement: Supplementary file 1 — Appendix 01 (PDF) [file pnas.2312030121.sapp.pdf]

Supplemental Materials: Statistical and Methods Appendices to the DICE-2023 Model[1]

December 28, 2023

Lint Barrage[2] and William Nordhaus[3]

Contents

Appendix A.	Supplemental figures and tables
Appendix B.	Overview of Changes in DICE-2023
Appendix C.	Real interest rates, the climate beta, and the discount rate
Appendix D.	DFAIR Model
Appendix E.	Abatement and backstop cost
Appendix F.	The damage function
Appendix G.	Modeling land-use and non-CO_2 GHGs
Appendix H.	Sources for data and concepts for DICE-2023
Appendix I.	Baseline emissions and carbon prices
Appendix J.	Projections of per capita GDP growth
Appendix K.	Computational and algorithmic aspects
Appendix L.	GAMS source code
Appendix M.	References

[1] These appendices provide supporting material for Lint Barrage and William Nordhaus, "Policies, Projections, and the Social Cost of Carbon: Results from the DICE-2023 Model," November 30, 2023, available at https://bit.ly/3TwJ5nO. They are not published and are for the information of readers as well as users of the DICE-2023 model. Please cite as "Supplemental Materials: Statistical and Methods Appendices to the DICE-2023 Model, December 28, 2023." See the main manuscript for acknowledgements. Further information on major topics is included in *Background Notes* on special topics, see (13), (20), (22), and (47) in the list of references in Appendix M.

[2] Associate Professor and Chair of Energy and Climate Economics, ETH Zurich.

[3] Sterling Professor of Economics, Yale University. Corresponding author, email: william.nordhaus@yale.edu.

Appendix A. Supplemental figures and tables[4]

	CO2 emissions, GtCO2/year			
Scenario	2020	2025	2050	2100
C/B optimal	42.9	42.9	37.1	15.9
T < 2°C	42.9	42.9	27.2	1.2
T < 1.5 °C	42.9	13.1	5.7	0.0
Alt damage	42.9	42.7	20.9	0.0
Paris extended	42.9	43.3	44.4	42.3
Base	42.9	44.9	54.6	75.7
R = 5%	42.8	42.5	42.2	37.6
R = 4%	44.1	43.9	39.3	28.9
R = 3%	45.6	45.3	33.5	15.4
R = 2%	46.8	46.7	22.5	0.0
R = 1%	46.8	46.9	19.2	0.0

Table A-1. Results for CO_2 emissions in different scenarios.
(a) Note that the 1.5 °C scenario has a catastrophic reduction in output to reduce emissions.

	CO2 concentrations, ppm				
Scenario	2020	2025	2050	2100	2150
C/B optimal	416.2	429.9	487.8	569.2	497.9
T < 2°C	416.2	429.9	474.7	474.7	437.9
Alt damage	416.2	429.8	466.7	458.5	401.0
Paris extended	416.2	430.1	501.3	652.5	763.5
Base	416.2	430.9	517.7	774.9	1,144.0
R = 5%	416.2	429.7	495.7	635.5	671.6
R = 4%	416.2	430.4	491.8	605.3	592.0
R = 3%	416.2	431.2	484.0	555.1	494.2
R = 2%	416.2	431.9	473.8	484.9	419.3
R = 1%	416.2	432.0	473.3	449.5	389.3

Table A-2. CO_2 concentrations, parts per million (ppm) by scenario

[4] The tables are updated to version DICE2023-b-4-3-10.

Scenario	Global temperature, °C relative to 1765				
	2020	2025	2050	2100	2150
C/B optimal	1.25	1.42	1.92	2.58	2.29
T < 2°C	1.25	1.42	1.85	2.00	1.86
Alt damage	1.25	1.42	1.81	1.89	1.58
Paris extended	1.25	1.43	2.01	3.00	3.61
Base	1.25	1.43	2.10	3.55	4.91
R = 5%	1.25	1.42	1.97	2.93	3.24
R = 4%	1.25	1.43	1.95	2.77	2.84
R = 3%	1.25	1.43	1.90	2.49	2.26
R = 2%	1.25	1.43	1.84	2.07	1.73
R = 1%	1.25	1.43	1.84	1.81	1.49

Table A-3. Global temperature increases under different scenarios

	Emissions control rate (%)					
	2020	2030	2040	2050	2060	2100
C/B optimal	5%	24%	31%	39%	46%	84%
T < 2°C	5%	24%	42%	55%	69%	99%
Alt damage	5%	24%	48%	65%	76%	100%
Paris extended	5%	13%	21%	27%	33%	57%
Base	5%	6%	8%	10%	12%	22%
R = 5%	5%	19%	23%	29%	34%	60%
R = 4%	5%	24%	29%	36%	42%	70%
R = 3%	5%	24%	39%	47%	54%	85%
R = 2%	5%	24%	48%	66%	73%	100%
R = 1%	5%	24%	48%	72%	90%	100%

Table A-4. Emissions control rate for CO_2 and abatable GHGs (percent of no control)

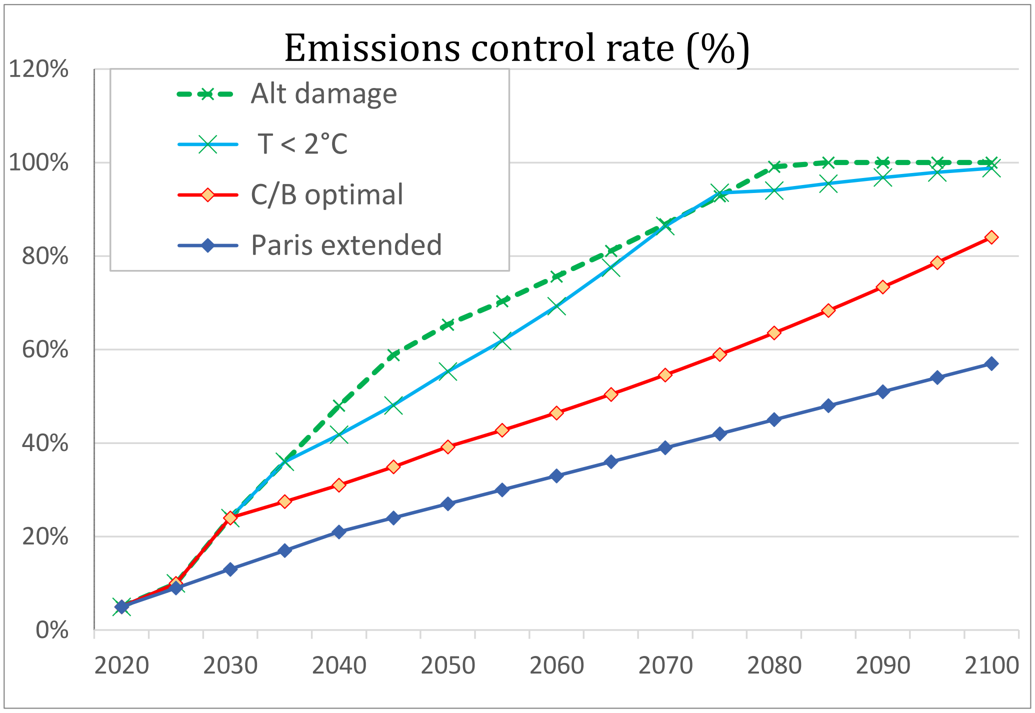

Figure A-1. Emissions control rate for CO_2 and abatable GHGs (percent of no control)

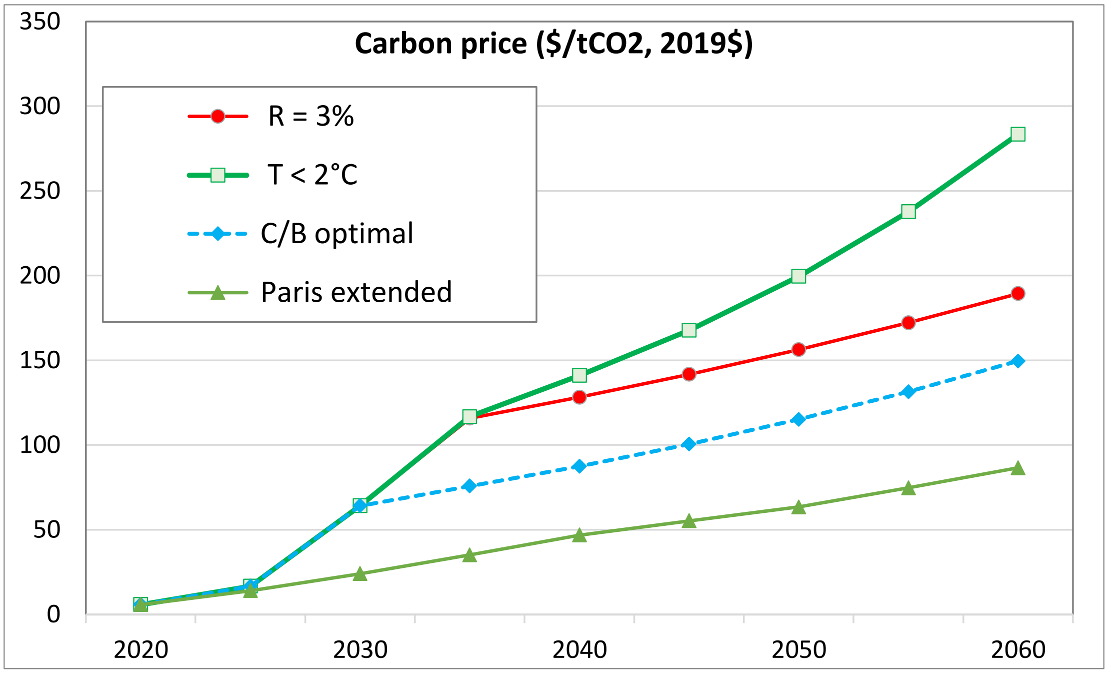

Figure A-2. Price of CO_2 emissions (2019 $/t$CO_2$)

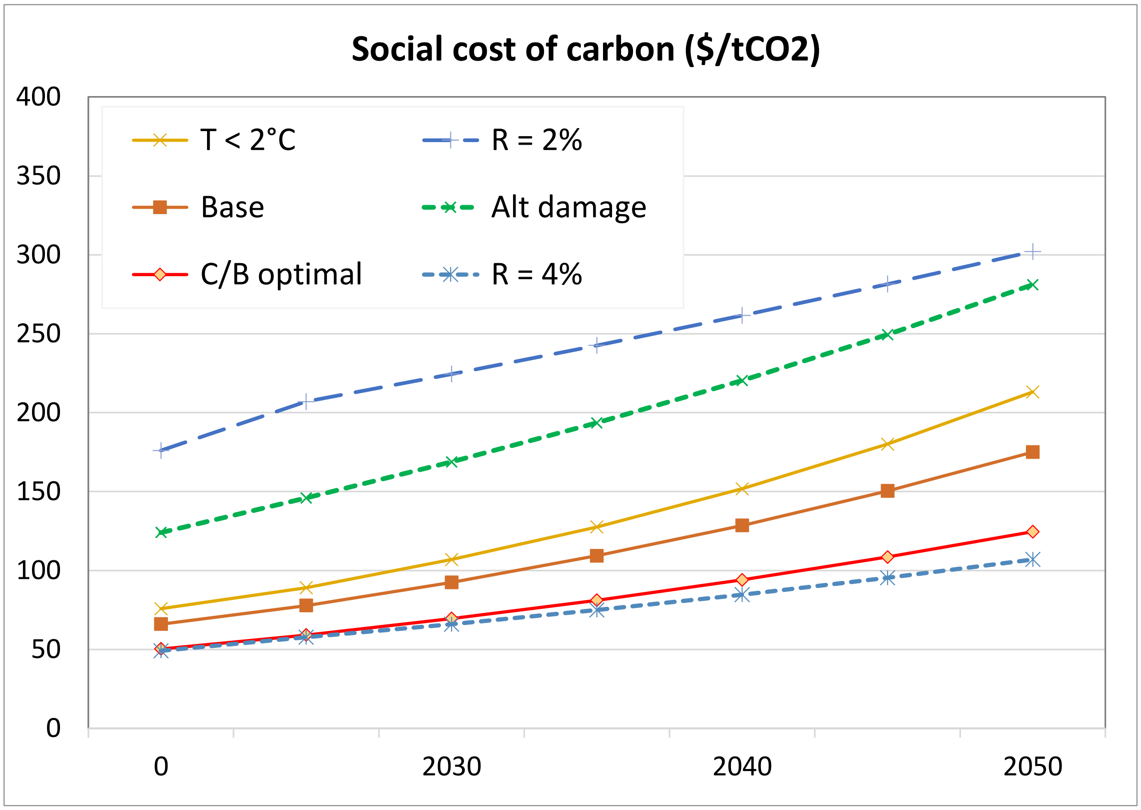

Figure A-3. Social cost of carbon, alternative scenarios (2019$/$tCO_2$)

Appendix B. Overview of Changes in DICE-2023

The current version of the model is **DICE2023-b-4-3-10.gms**, October 16, 2023. This is implemented in the GAMS modeling system (see Appendix L). The full description is discussed below. This note provides an overview of the revisions between DICE-2016R3 and DICE2023-b-4-3-10.gms. A discussion of the data sources and methods is provided in these Appendices.

A.1. Major changes

There are four major revisions in DICE-2023. The first and most significant change in DICE-2023 are new carbon and climate cycles. We adopted the FAIR model (1) for both the carbon and climate modules. The carbon cycle is methodologically different from earlier approaches in including saturation of the non-atmospheric reservoirs. The climate module is a two-box model, similar to D2016 but with different parameters. The TSC and ESC are largely unchanged from D2016 but brought in line with the latest IPCC Assessment Report (2). The parameters of the model are shown in Appendix D. Note that the major adjustment from the standard FAIR model is to determine the initial conditions for the four reservoirs of the carbon cycle for 2020 and to calibrate the climate parameters.

A second methodological change is the treatment of discounting. We review data on real interest rates and the return to capital. Moreover, we explicitly account for investment risk in climate abatement. Data on real returns are drawn from market interest rates and equity yields as well as data on the rates of return to non-financial capital from the US Bureau of Economic Analysis. We incorporate an explicit risk premium on risky capital and a climate-investment beta of 0.5 from (3). Additionally, we incorporate an estimate of the precautionary effect due to uncertainty about future growth. On the basis of these revisions, we estimate that the real return on climate investments is slightly lower than in D2016 in the near term (4.5%/year in DICE-2023 v 5.0%/year in DICE-2016 for 2020), and significantly lower in the longer run.

A third major change is the damage function. We update the damage function in three ways. First, we extend the analysis of (4) by adding studies of global aggregate climate change impacts that have been published in recent years, based on a review (5). Second, we add a new component to account for tipping points based on (6). Third, we add a judgmental adjustment factor to reflect non-monetarized impacts as explained in Appendix F. The updated damage estimates take the form of a quadratic damage function with a 3.1% loss in global output from 3 °C warming over preindustrial temperatures, up from 1.6% in DICE-2016. We emphasize that this estimate is subject to ongoing further refinements of the different components.

A fourth change is to add abatement for forcings other than industrial CO_2. The procedure is to (i) estimate current and future GHG emissions and forcings; (ii) convert them to CO_2-equivalent forcings; (iii) determine the fractions that are abatable; and (iv) use the estimates of cost of abatement for those from the existing literature. The estimates

for (iii) and (iv) are from (7). Total abatable emissions are equal to all CO_2 emissions plus abatable non-CO_2 GHG (in CO_2 equivalent emissions). The abatement cost and control will then be applied uniformly to total abatable emissions. This version implies that 95% of forcings are abatable compared to 80% of forcings in the D2016 version. See the discussion in Appendix G.

A.2 Other changes

Data

This section provides a summary of data changes and revisions. Each period is five years, with further details in the following appendices. A period is calculated as the average of the five years centered on the given year. Thus, “2020” is the average for 2018-2022. Note that the stocks are at the end of period, so “2020” is conceptually end-2022 for stocks.

All important input parameters are updated on the basis of the most recent information, usually through 2022. The historical and contemporaneous economic data include updates for world output, population, CO_2 emissions, non-CO_2 greenhouse gas forcings, and the emissions-output ratio (σ, sigma). Data are all stated in 2019 US international dollars measured at PPP exchange rates. Note that the initial period (2020) has a major anomaly because it contains the highly depressed pandemic year of 2020. We have included actual data for 2020, but exclusion would make little difference for the projections.

Projections of these data are as follows: Population projections are from the UN (https://population.un.org/wpp/). Output is measured in 2019 PPP US$, with the historical and current estimates from the IMF and the World Bank being virtually identical. Projections of output per capita are based on several studies (8-11). The no-controls emissions-output ratio for industrial CO_2 is assumed to continue to decline at its historical rate of 1.5% per year. The CO_2 emissions from land use are from IPCC AR6 (Physical Science, (2)), and projections of non-CO_2 GHGs are on the basis of projections from MAGICC6.

The backstop technology is derived from the simulations of the ENGAGE project (12). It has a backstop cost that is similar to estimates of other model comparisons and has an estimated decline in the zero-emissions carbon price of 1% per year until 2050. This is further discussed in Appendix E.

Concepts and functions

The abatement cost function is derived from the backstop cost for industrial CO_2 (see the last paragraph and Appendix E). The functional form for abatement cost is the same as in D2016.

The "baseline" policy is changed from earlier versions to include a low level of carbon prices and regulation, and emissions are therefore lower than the no-control level. In current policy, it is assumed that emissions are initially about 5% lower than the no-control level, which is equivalent to a carbon price of $6 per ton, and that the carbon price will grow by 2.5% per year. This is half due to carbon pricing and half due to regulations.

Major data and information about the climate and carbon cycle are from the sixth assessment report for science IPCC AR6 (Physical Science, (2)). Additionally, data on scenarios are drawn from the IPCC socio-economic storylines (SSPs)[5]. We particularly referred to SSP2, which is characterized as a "middle of the road" scenario. For calibration purposes, we also used IPCC Representative Concentration Pathways (RCPs), particularly RCP45 and RCP85, IPCC AR6 (Physical Science (2)).

The model is scaled so that utility in the baseline run has marginal value of the objective function of 1 for a change of 1 unit of consumption in 2019$. The additive scaling was set so that the present value of consumption equals the objective function for a base run of 400 years.

[5] Institute for Applied Systems Analysis (IIASA), "Shared Socioeconomic Pathways Scenario Database (SSP)." URL (accessed January 2022): https://iiasa.ac.at/models-tools-data/ssp

Appendix C. Real interest rates, the climate beta, and the discount rate

This appendix summarizes the background, data, and analysis on discounting in the DICE-2023 model. A more detailed discussion is contained in a more detailed discussion in *Background Notes on Discounting* (13).

C.1. Trends in real interest rates

We have reviewed historical data on returns for major assets classes in the US for the 1927- 2022 period. Note that these are total returns including dividends, interest, and capital gains or losses. Additionally, we have estimated the rate of return (total earnings on capital, i.e., profits plus interest) as a percentage of the current replacement cost of the net stock of fixed assets of the US corporate sector. These data are provided in the *Background Notes on Discounting* (13).

Based on these findings, we conclude the following:

1. The short-run risk-free return (measured as the real return on short Treasury securities) has averaged close to zero per year for most of the last century, although it has been lower in recent years.
2. The long-run risk-free rate of return (measured on 10-year Treasury bonds) has averaged around 2% per year over the total period, although it has been sharply lower in the last decade. The real interest rate on 30-year TIPS has a shorter period and has recently risen back to the earlier pre-financial crisis level of about 2% per year.
3. The best estimate of the after-tax real return on capital (measured in the US corporate sector) has been 7 – 9% per year over the last half-century. Unlike financial returns, there has been no major change in these returns in the last two decades.
4. Corporate equities are currently unleveraged with respect to fixed-interest type assets. We therefore treat corporate equities as unleveraged.

C.2. The capital premium

We define the "capital premium" as the difference between the expected return on aggregate economy-wide assets and the long-term risk-free rate of return. Based on the current balance sheet of the corporate sector, we assume that the capital premium is the same as the well-studied equity premium. We observe these data only for the US corporate sector and assume that these values apply to the entire global financial structure. Based on the estimates above, we assume that the rate of return on risky assets in the US is 7% per year and has been relatively stable. That rate applies not only to financial returns but also to corporate capital. Based on the estimate of a risk-free long-run rate of return of 2% per year, we calculate the capital premium to be 5% per year.

C.3. Treatment of discounting in DICE-2023

Previous versions of DICE used different approaches to discounting. Upon the advice of modelers and readers, we have revised the treatment to employ an approach known as the "certainty equivalent" rate of return. This approach has been developed using suggestions of William Hogan, theoretical approaches developed by Gollier (particularly (14)), recommendations of the National Academy committee (15), and empirical implementation of the correction for growth uncertainty by (11), hereafter NPP.

This summary provides a brief description of the revised approach. A full discussion of the approach is contained in the *Background Notes on Discounting* (13). This procedure is followed for all scenarios except the constant discount-rate scenarios, which have rates equal to 1%/year, …, 5%/year. For the constant discount-rate scenarios, we set ρ equal to the relevant discount rate (e.g., 1%/year) and then set all other parameters at close to zero (for computational reasons > 0). All variables are defined in the accompanying footnote.[6] Discounting continues to follow the approach of the Ramsey-Cass-Koopmans growth model in determining real rates of return. In this approach, the continuous-time equilibrium deterministic long-run rate average of return from 0 to T $\left(\tilde{R}_T\right)$ is given by the pure rate of time preference (ρ) plus the product of the deterministic average growth rate of per capita consumption from t = 0 to T $\left(\tilde{g}_T\right)$ times the elasticity of the marginal utility of consumption (φ). Note that the "~" over a variable indicates a deterministic concept.

$$\text{(C.1)} \quad \tilde{R}_T = \rho + \varphi \tilde{g}_T$$

In DICE-2023, φ represents the extent of substitutability of the consumption of different years or generations. In many applications, the consumption elasticity is also assumed to equal the relative rate of risk aversion. This is not assumed in the DICE treatment of discounting. That assumption would lead to a capital risk premium that is far below the observed rate discussed in the literature on the equity-premium puzzle (also see (13)). Instead, we rely on the CAPM estimates of the capital premium.

The modeling relies upon discount rates and their associated discount factors to calculate present values, optimal policies, and variables such as the social cost of carbon. The "discount factor," D_T, is the factor applied to future values to obtain the present value of a value in time T discounted back to time 0. In a deterministic framework, the discount factor is the product of the one-period discount factors. In this discussion, r_t are period-to-period rates of return from period $(t-1)$ to t, while R_T are long rates of return from period 0 to period T (all in compound annual rates).

---

[6] The variables in the analysis are the following. All rates are average annual returns, and all time variables are per year. R_T = discount rate from 0 to T; r_t = discount rate from t-1 to t ; R_T^f, R_T^{CLIM} = risk-free and climate discount rates ; ρ = pure rate of time preference; π = capital premium ; P_T = precautionary effect rate from 0 to T; φ = elasticity of utility with respect to consumption ; ρ_T^* = risk-adjusted rate of time preference ; β^{CLIM} = climate beta ; g_T = average annual growth of per capita consumption from 0 to T ; σ_C^2 = variance of trend growth rate of per capita consumption ; $\tilde{x}_T$ = deterministic version of variable x_T.

$$(C.2)\quad D_T = \left[\frac{1}{\left(1+R_T\right)^T}\right] = \left[\frac{1}{(1+r_1)}\right]\left[\frac{1}{(1+r_2)}\right]\cdots\left[\frac{1}{(1+r_T)}\right]$$

Because of uncertainty about future growth, the *expected* discount factor will differ from the deterministic discount factor by a term called the "precautionary effect." The precautionary effect in the near term is small, but with long horizons, the impact of *uncertain trend growth* can be substantial,[7] and for that reason the precautionary effect has a major impact on climate policy.

In the approach taken here, we assume that the major uncertainty is about the long-run trend rate of growth of per capita consumption, which is assumed to follow a normal distribution with known mean and variance $\sigma_C{}^2$. The precautionary component for a normal distribution of trend growth rates is given by

$$(C.3)\quad P_T = -½\,\sigma_C{}^2\varphi^2 T$$

where P_T = is the precautionary effect from time *0* to *T*. In this process, where there is uncertainty about the trend rate of growth, the precautionary effect will be larger as the length of period increases because the variance of log consumption increases. For a deterministic model like DICE, we therefore correct the deterministic Ramsey equation to reflect growth uncertainty through adding the precautionary effect.

This procedure generates a sequence of "certainty-equivalent discount rates." This term is used to designate the single discount rate delivering the same discount factor as the expected value from the distribution of uncertain future discount rates (11, p. 1019). From (C.1) and (C.3), the certainty-equivalent risk-free discount rates (R_T^f) are given by (C.4):[8]

$$(C.4)\quad R_T^f = \rho + \varphi\,\tilde{g}_T - ½\,\sigma_C{}^2\varphi^2\,T$$

To calculate the precautionary effect, we examine two procedures. The first is based on the calculations of NPP. These take estimated future growth rates from their Monte Carlo draws and the implied future interest-rate structure to estimate numerically the precautionary component. A second approach takes the standard formula in (C.3) for the precautionary effect from a model with a normal distribution of the trend growth rate. The two approaches give reasonably similar estimates of the precautionary effect, and we therefore take equation (C.3) as computationally simpler and easier to implement and test.

---

[7] An important alternative assumption is where the growth rate of consumption is uncertain with a constant mean but independently distributed across time periods, in which case the precautionary effect is constant over time (see, e.g., (15)).

[8] See, e.g., (16) equation (1) or (14) equation (37), where we note in reference to the latter that our approach to discounting uses the CAPM rather than CCAPM approach for adjustments to the risk profile of climate investments, as discussed below.

The key parameters of the precautionary effect are the variance of the consumption growth rate and the consumption elasticity. The variance is estimated in several studies (8--9). The studies have estimates of the standard deviation of trend growth of per capita consumption to 2100 in the range of 1.0% to 1.2% per year. For our modeling, we assume that consumption per capita is a process in which trend growth is normally distributed with a standard deviation of 1 percentage point per year. So, the uncertainty is in the *trend growth rate,* not in the *level* of log consumption. That is, the expected value of the growth rate of per capita GDP from 2020 to 2100 might be a mean of 2% per year, with a mean-plus-one-sigma of 3% per year. The *Background Notes on Discounting* (13) describes the calculation of the precautionary effect in detail. Appendix J provides updated estimates of future economic growth.

In making calculations for DICE-2023, we rely on two components of the discount rate: a risk-free rate and an adjustment for investment risk. A broad consensus exists that the risk-free real return on investment is in the range of 0 to 2% per year over the last century. We take 2% per year to be the rate for long-term risk-free investments, which is the rate that has prevailed over the last century or so except for the most recent period.

Empirical evidence indicates that the return to risky assets (such as corporate capital or an unleveraged portfolio of corporate equities) is substantially higher than the risk-free rate. For example, the post-tax average rate of return on US corporate capital has averaged around 7% per year over the period from 1948 to 2022. The real return on a deleveraged portfolio of large US public corporations was 6% per year for the same period. The underlying data are presented in the *Background Notes on Discounting* (13).

At this point, we confront the "equity premium puzzle." This puzzle is that the volatility of consumption cannot rationalize the high risk premium (of 5% per year in our estimates) within the standard model (the C-CAPM model). Most studies examine the equity premium, but the puzzle remains for capital as well as equity. Given the failure of the C-CAPM model, we adopt the estimates from the CAPM model, which examines the correlation of investment risks with the market risk rather than the consumption risk. This leads to the assumption of a risk premium of 5% per year in the DICE-2023 model.

In the DICE-2023 specification, the discount rate includes an adjustment for the non-diversifiable risk of climate investments. Risky climate investments, primarily those to reduce emissions, are introduced through the concept of the climate beta. The climate beta measures the extent to which climate investments (such as renewable power) share the non-diversifiable risk characteristics of the economy's aggregate investments. A climate beta of zero indicates that the risks on climate investments are uncorrelated with market returns; a climate beta of one indicates that climate investments have risk properties similar to those of the aggregate economy. We take our estimate of the climate beta from (3), which estimates a long-run climate beta of 0.5, so one with an intermediate correlation with market risks. A more extensive discussion of the climate beta and the reason for our estimate is given in the *Background Notes on Discounting* (13).

For our purposes, we assume that the risk-free long-term rate is 2% per year and the capital risk premium is π = 5% per year. With a climate beta of 0.5, this implies a near-term risk-adjusted certainty-equivalent discount rate on climate investments of 2% + 0.5 x 5% = 4.5% per year. We note that the precautionary adjustment is taken to be zero in this illustrative calculation as near-term consumption growth trend uncertainty is minimal.

To calibrate the model requires estimates of φ and ρ. These are estimated by first calculating the deterministic risk-free rate of return from (C.1) above (where we note that using the certainty-equivalent rate equation (C.4) would yield equivalent results again due to the small level of near-term growth uncertainty). We constrain $\rho \geq 0.1\%$ per year to ensure long-run convergence and incorporate the DICE estimates of near-term growth in per capita consumption of 2% per year. Solving for φ gives the following:

$$(C.5) \quad \varphi = \left(R^f_{2020} - \rho\right)/g_{2020} = (0.02 - 0.001)/0.02 = 0.95$$

This then implies that the average annual discount rate on climate investments (R_T^{CLIM}) from 0 to T is:

$$(C.6) \quad R_T^{CLIM} = \rho + \varphi\tilde{g}_T - ½\varphi^2{\sigma_C}^2 T + \beta^{CLIM}\pi$$

Substituting the values of ρ, φ, $\tilde{g}_0$, ${\sigma_C}^2$, π, and β^{CLIM} into (C.6), this calculation gives an estimate of 4.5% per year for T = 0. In order to implement (C.6) in DICE, we replace the pure rate of social time preference in equation (C.1) with a risk-adjusted rate of time preference designated by $\rho_T^* = \rho - ½\varphi^2{\sigma_C}^2 T + \beta^{CLIM}\pi$. Note that these equations are for the continuous-time version and must be adjusted for discrete time.

Appendix D. DFAIR Model[9]

The carbon cycle and climate model are key components of any IAM. DICE-2023 has made a major change to the treatment of these modules, particularly the carbon cycle. Earlier versions in many models have used a three-box linear carbon cycle. While this approach seemed acceptable as a simplification, recent studies and advances have shown that a simple modification could allow the model to reflect the important finding that the ability of non-atmospheric sinks to absorb CO_2 declined with higher emissions. (See (15) for a discussion.)

The extent of dependence was discussed as early as 1957 in (17). The latest and most extensive multi-model comparison was (18). This showed that the atmospheric retention at 100 years after a pulse would be 30% for a pulse of 100 GtC compared to 70% for a pulse of 5000 GtC.

While this has been known for many years, a simple approach has become available over the last decade. The new module is called the DFAIR module, where DFAIR is the DICE version of the FAIR or Finite Amplitude Impulse-Response model developed by Millar et al. (1). The DFAIR module follows the Millar approach closely. The FAIR model started with a linear four-pool time-constant impulse-response model of the CO_2 concentration response to emissions. While the pools may have names ("permanent," "long," etc.), they are not physical reservoirs but are reduced-form dynamic equations. Note that the response of concentrations is independent of the size of the emissions in the starting model.

In today's detailed process models, the atmospheric retention declines with higher emissions. The FAIR model captures this by adding a single time-varying parameter (α_t) to each of the time constants of the linear carbon cycle model. This factor leads to lower atmospheric retention with larger cumulative emissions of CO_2. For more details, see (1). An early incorporation of FAIR into DICE-type models was undertaken by (19).

Adoption of the FAIR model required three steps. First, the coding (which was for annual or very short times steps in Millar) needed to be transformed to the five-year time step in DICE. Second, we needed to ensure that the DICE version tracked both Millar and (18) sufficiently closely. Third, the FAIR model requires initial conditions for 2020 that are path-dependent and therefore needed to be calculated for the emissions history and for 2020 concentrations.

The first step of moving to the five-year time step was straightforward as model parameters are either fractions or years and do not need recalibration. The only parametric change was to change the equilibrium and transient sensitivity coefficients of the impact of doubling CO_2 concentrations on global temperature (ESC and TSC). The

[9] For a full discussion of the results and methods, see *Background Notes on DFAIR* (13).

Millar version set the ESC at 2.75 °C, while the DICE version is 3.0 °C. One change is that the forcings per CO_2 doubling was set to 3.93 W/m2 instead of 3.74 W/m2 in Millar. This change matches the best estimates from AR6. These two changes required recalibrating the other FAIR parameters. The was done by minimizing a quadratic loss function subject to changing the parameters minimally. The results are seen in Table D-1.

The second step – testing the model – was the most difficult. While (1) demonstrates, among other things, the ability of the FAIR model to closely match future temperature projections for different emissions scenarios of richer climate models, we wanted to consider three comparisons of particular interest.

The first comparison examined the impact of a 100 GtC pulse in 2010, as in the multi-model comparison study of Joos et al. (18). The Joos study has been the basis of critiques of prior DICE vintages by Dietz et al. (19). Figure D-1 shows several impulse-response curves as follows: The projected temperature response of the DFAIR model (blue line with circles, modified in line with the relevant experimental conditions as described in (20)); the Joos et al. multi-model mean (red line with x's) plus and minus two standard deviations (grey area); the CMIP5 best-fit line from Dietz et al. (19) (green line); and the impulse response in the DICE-2016 model (grey dashed line) as estimated by (19). The results indicate that the DFAIR model successfully addresses the concern that prior vintages of DICE exhibited excessive warming inertia in response to emissions pulses compared to recent climate model estimates as shown in (18). As in the original FAIR model of Millar, the temperature response is slightly low initially and slightly high in the longer run, but overall, the fit appears satisfactory, especially along with the other model targets considered.

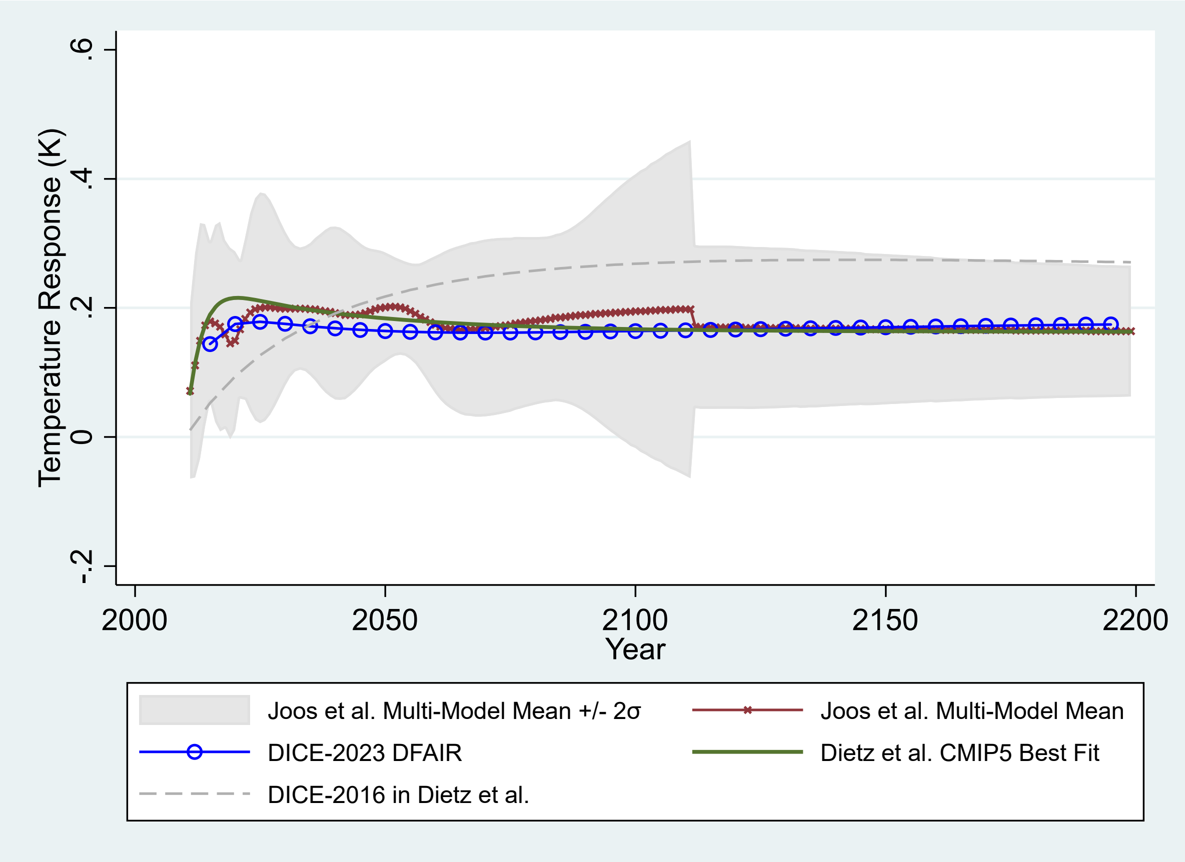

Figure D-1: Temperature responses to a 100 GtC pulse in 2010 by the modified DICE-2023 DFAIR model, the multi-model mean plus/minus two standard deviations from Joos et al. (18), the CMIP5 best fit line from Dietz et al. (19), and the DICE-2016 response as estimated by (19).

The second set of tests considered were the impacts of pulses of 100 GtC and 5000 GtC from pre-industrial conditions, also as in (18). The results (see the *Background Notes on DFAIR* (20)) again indicated that the FAIR model underprojected concentrations at short horizons (< 60 years for both) and overpredicted at longer horizons (>200 years), with mixed results between 60 years and 200 years after the pulse.

The third experiment examined the results of using the FAIR model to project CO_2 concentrations using estimated CO_2 emissions from 1765 on. To test history, we created the "1765 model," which ran starting in 1765 with zero values for initial values for the carbon reservoirs and temperatures (all relative to 1765). We then ran with estimated CO_2 emissions. The historical test also showed that FAIR underestimated concentrations, but by a small amount. For 2020, the FAIR projection was 417 ppm compared to the actual estimated value of 420 ppm. Given these findings, the parameters of the carbon cycle in Millar were judged accurate for the DICE approach.

To complete the calibration of the DICE-2023 model requires setting initial conditions of the FAIR module for 2020. To accomplish this, we performed a slight

modification of the 1765 model described in the last paragraph. The objective of the calibration was to ensure that the simulation from 1765 to 2020 produces a 2020 calculation that matches the actual value of concentrations in 2020. After some experimentation, we decided the simplest approach was to increase historical emissions before 1960 by 10%. Doing this led to concentrations matching history closely from 1960 to 2020. We then took the stocks in each of the four reservoirs in 2020 in the modified 1765 model as initial conditions for the standard DFAIR model. We checked to see if the splice was smooth, and it proved satisfactory.

Overall, the structure and performance of the DFAIR module seems to be satisfactory. The one minor issue is the slight underprediction of the atmospheric concentrations at the short end as well as some second-order differences from full earth-system models. The impact appears to be minimal, so refinements will be left to further research. Again, for a fuller discussion, see *Background Notes on DFAIR* (20).

Parameters	Millar (orig)	Dietz	DFAIR
emshare0	0.2173	0.2173	0.2173
emshare1	0.2240	0.2240	0.2240
emshare2	0.2824	0.2824	0.2824
emshare3	0.2763	0.2763	0.2763
tau0	1,000,000	1,000,000	1,000,000
tau1	394.4000	394.4000	394.4000
tau2	36.5300	36.5300	36.5300
tau3	4.3040	4.3040	4.3040
teq1	0.3300	0.3300	0.3240
teq2	0.4100	0.4100	0.4400
d1	239.0000	239.0000	236.0000
d2	4.1000	4.1000	4.0700
IRF0	32.4000	34.4000	32.4000
irC	0.0190	0.0190	0.0190
irT	4.1650	4.1650	4.1650
fco22x	3.7400	4.2000	3.9300
mat0	588.0000	588.0000	588.0000

Table D-1. Comparison of the parameters of the different FAIR models

The parameters are for the equations of the carbon cycle and climate module. Entries in light color in last column are different from Millar et al. (1). Definition of parameters is in Table D-2.

Variable	Units	Definition
emshare0	Fraction	Geological re-absorption
emshare1	Fraction	Deep ocean invasion/equilibration
emshare2	Fraction	Biospheric uptake/ocean thermocline invasion
emshare3	Fraction	Rapid biospheric uptake/ocean mixed-layer invasion
tau0	Year	Geological re-absorption
tau1	Year	Deep ocean invasion/equilibration
tau2	Year	Biospheric uptake/ocean thermocline invasion
tau3	Year	Rapid biospheric uptake/ocean mixed-layer invasion
teq1	$KW^{-1}m2$	Thermal equilibration of deep ocean
teq2	KW-1m2	Thermal adjustment of upper ocean
d1	Year	Thermal equilibration of deep ocean
d2	Year	Thermal adjustment of upper ocean
IRF0	Year	Preindustrial iIRF100
irC	$YearGtC^{-1}$	Increase in iIRF100 with cumulative carbon uptake
irT	$YearK^{-1}$	Increase in iIRF100 with warming
fco22x	$KW^{-1}m2$	Forcings for CO2 doubling
mat0	GtC	Initial carbon stock

Table D-2. Definition of variables for DFAIR and FAIR models

Appendix E. Abatement and backstop cost

Two important parameters, but ones without a strong empirical support, are the initial the price of the backstop technology $(\overline{p})$ and its decline rate (g_{pback}). For DICE modeling, the concept of the backstop price measures the carbon price necessary to attain zero net greenhouse gas emissions. This parameter can be measured indirectly from engineering or econometric abatement cost function. However, the estimates are particularly speculative at high rates of emissions control, particularly as economies approach zero net emissions, because the carbon prices and control rates are well outside historical experience.

One useful source for the estimate of the backstop cost is model-comparison studies, particularly ones that look at high carbon prices and high control rates. We undertook a statistical analysis from results of the ENGAGE study (12, 21) which indicates a median backstop price of \$515/t CO_2 in 2019\$ in 2050, which is the earliest year that most models can reach zero net emissions. This will be used as the backstop cost for 2050. Models assume improvements in technology lead to a decline in the carbon price needed to attain zero emissions. We assume that the backstop technology cost declines 1%/year from 2020 to 2050, and then 0.1%/year thereafter. Using this rate for 2020 to 2050 yields a backstop price of \$695/t CO_2 in 2020 in 2019\$, which is used in DICE-2023.

The DICE model uses a log-linear abatement cost function of the form:

$A(t) = Q(t)\theta_1(t)\mu(t)^{\theta_2}$, where $A(t)$= abatement cost, $Q(t)$ = output, $\mu(t)$ = emissions control rate, and $\theta_1(t)$ and θ_2 are parameters of the abatement cost function.

We add a commentary on the estimates. First, the exponent of the abatement cost function, θ_2, is estimated from abatement-cost estimates in several models and approaches. It is generally estimated from an equation of the logarithm of the cost/output ratio on the emissions control rate. The estimates always produce a highly convex function with an exponent between 2 and 3, and we take a coefficient of 2.6 as a central value. Second, the cost of the backstop technology (measured as the carbon price at which net emissions of all abatable greenhouse gases are zero) is calculated from different model runs that generate zero net emissions by around 2050. Based primarily on (12), we estimate the cost of the zero-net-emissions-parameter, $\theta_1(t)$, to be around 11% of output in 2020 and 5.1% of output in 2050. We emphasize that the abatement cost parameter $\theta_1(t)$ refers to the total abatable emissions-output ratio (that is, including non-CO2 abatable emissions). By assumption, the model does not allow zero net emissions before 2070 or negative emissions before 2120.

Looking at studies of low- and zero-carbon technologies, the estimated price of the backstop appears higher than many current estimates. However, those optimistic prices are generally about unproven technologies or ones that have not been developed at the massive scale required to reach zero emissions by mid-century. Note in any case that the estimates of the abatement cost function have little effect on the social cost of carbon, although they have a major impact on the cost of attaining ambitious climate targets.

Appendix F. The Damage Function

DICE-2023 maintains the structure of the damage function from DICE-2016 but updates its quantification in three ways: We update the literature synthesis of Nordhaus and Moffat (4), we add a term for tipping-point impacts based on estimates from (6), and we increase the judgmental adjustment term to reflect omitted impacts, uncertainty, and further considerations, as described below.

One important point is that we rebase all temperature estimates to the "preindustrial" global mean temperature, which we take to be the period centered on 1765. This rebasing leads to a mean temperature in the DICE calculations that is about 0.4 °C higher than the earlier basis of 1920 – 40. The rebasing also requires adjusting damage estimates in the literature, which use different temperature baselines.[10] The *Background Notes on Damages* (22) provides a detailed discussion of the temperature baselines and adjustments made for each study used in the DICE-2023 damage function. Note that DICE-2023 quantification of climate damages includes estimates of the value of the impacts of non-market goods and services where available. Income levels are initialized based on conventional output measures. The inclusion of non-market values in income levels would affect the level of total income, but because of the normalization of market output, it would not have a significant effect on the key results in our framework.

<u>F.1 Updating Nordhaus and Moffat (4)</u>

The core of the DICE-2016 damage function was based on a review of estimates of global aggregate climate change impacts by Nordhaus and Moffat (4, "NM"). Their preferred specification aggregated 38 estimates from the literature in a weighted quantile regression. We update this analysis in two ways. First, we add 18 new estimates of aggregate climate change impacts from 11 studies published since the NM review.

The update is based on (5) that reviewed alternative methodologies and studies quantifying aggregate climate change impacts.[11] Second, we make a few adjustments to

[10] For example, an estimate of 3% GDP loss at 3.00 °C warming over 1985-2005 levels may appear similar to our benchmark estimate. However, the DICE-2023 damage estimate would be 5.4% GDP loss at the corresponding 3.95 °C warming over preindustrial temperatures.

[11] We specifically consider the studies listed in their Supplementary Materials Table (Section 3). Among the studies listed in this table, we focus on new bottom-up and top-down aggregate damage assessments. That is, we do not review studies which use prior impact estimates to propose new damage functions but do not themselves produce new climate change impact estimates. We further limit the sample to studies that are published and provide estimates of aggregate climate change impacts at the global level. We exclude one study for which we were unable to replicate the estimates, leaving us with 11 studies (23-33).

the original NM data, specifically changing two weights and correcting one temperature level.[12]

Figure F-1 displays a scatter plots of the original NM impact estimates (circles) and the new additions (squares) along with results from an OLS regression of impacts on temperature change squared (relative to 1920-40 levels, quadratic regressions without a constant or linear term, restricted to estimates for warming of less than 5°C).

The updated preferred estimates from the weighted quantile regression imply climate change damages of 2.16% of global GDP for 3 °C warming over 1920-40 global mean temperature, up from 1.63% in the original NM study.[13] Assuming 0.4 °C warming in 1920-40 relative to preindustrial temperatures and given a quadratic damage function, the updated estimates imply 1.562% damages at 3 °C warming over preindustrial temperatures.

F.2 Adding Damages from Major Tipping Points

Authors (6) present a comprehensive review and synthesis of impacts from major tipping points such as melting of the Greenland Ice Sheet. Their benchmark estimates imply an additional loss of global output of 1% from 3 °C warming over preindustrial levels due to tipping points. Because tipping points are generally omitted from the studies which form the basis of the benchmark damage estimate, we add these estimated costs to our damage estimate. This adjustment is subject to further review.

F.3 Updated Judgmental Adjustment Factor

DICE-2016 and earlier versions of the model included a "judgmental adjustment factor" that would incorporate omitted impacts, particularly those that were difficult to monetize. This was described as follows: "Current studies generally omit several important factors (the economic value of losses from biodiversity, ocean acidification, and political reactions), extreme events (sea-level rise, changes in ocean circulation, and accelerated climate change), impacts that are inherently difficult to model (catastrophic events and very long term warming), and uncertainty (of virtually all components from economic growth to damages)" (37).

[12] One change is to set the weight on (34)'s damage estimates for 10C warming to zero (instead of 0.1). The other adjusts the weight on (35) from 1 to 0.25 to reflect the fact that the updated review includes a newer version (23). Finally, we adjust the temperature for (36) from 1.92C warming to 1.52C to convert to warming relative to 1920-40 levels.

[13] Further details on the estimates used and weights attached thereto are provided in the *Background Notes on Damages* (22).

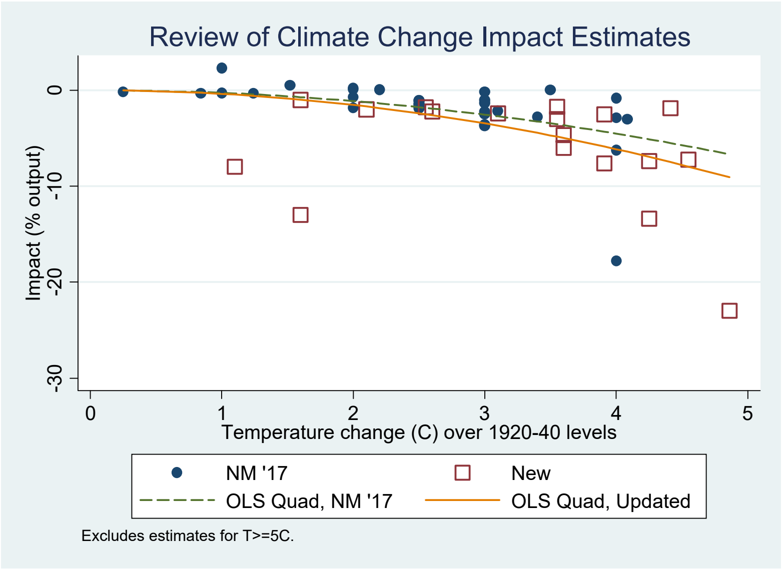

Figure F-1: Literature estimates of climate change impacts

In reviewing the literature since the NM study, we conclude that the factors cited above (with the exception of tipping points) largely continue to elude damage modelers. We put our judgmental adjustment for excluded impacts at 0.5% of output at 3 °C warming. This adjustment reflects three considerations:

(i) The list of unmonetized potential impacts listed above continues to remain of concern and incompletely understood.

(ii) The potential impact of uncertainty continues to be a thorny question, particularly fat-tailed events that are incompletely understood at present. Significant uncertainties are associated with many aspects of integrated assessment models (38). While our discount rate is risk-adjusted, several studies have found that additional upward adjustments of the social cost of carbon may be warranted due to different types of uncertainty (39-43).

(iii) We have not yet incorporated all recent climate change impacts research in our synthesis of aggregate damage estimates. The empirical literature has made significant advances in quantifying certain climate impacts. As an important example, (44) find that prior studies may substantially underestimate temperature-related mortality impacts from future warming.[14] Some other recent reviews of the literature also argue for higher overall damages than our own synthesis (46). These considerations motivate the updated judgmental adjustment in our damage function.

[14] On the other hand, a similarly comprehensive global analysis (45) indicates that some influential prior estimates of the impacts of energy consumption on future warming may be exaggerated. Formally accounting for these types of new studies would require a comprehensive sector-by-sector literature synthesis, which is beyond the scope of this study.

Appendix G. Modeling land-use CO_2 and non- CO_2 GHGs

The prior version of DICE had two sources of CO_2 emissions (industrial and land) as well as other GHGs included as "other forcings." Only industrial CO_2 was included in abatable emissions.[15]

In the new DICE-2023, all CO_2 emissions and a substantial fraction of non- CO_2 GHGs are abatable. The abatable fraction of non-CO_2 GHG emissions is limited to long-lived GHGs and, based on (7), is 65% of total CO_2 equivalent emissions. The important assumption then is that "abatable emissions" are all CO_2 emissions (from industrial and land-use sources) and 65% of other well-mixed GHGs.

We aggregate the three sources (industrial CO_2, land-use CO_2, and other abatable GHGs) as follows: land-use emissions are simply added to industrial emissions on the basis of current and future CO_2 emissions. Land-use emissions are from AR6 data and are assumed to decline 2% per year. Industrial emissions are from EDGAR data and are assumed to decline at 1.5% a year relative to global output. For other non- CO_2 GHGs, we use radiative forcings for well-mixed GHG less for CO_2 from RCP_6 from MAGICC (January 2022). These forcings are 1.03 w/m2 in 2020 and rise slightly then decline to 1.02 w/m2 in 2100.

Both the abatement cost function and the emissions control rate are assumed to be the same for industrial CO_2 emissions, land-use, and non- CO_2 emissions. Though simplified, this modeling approach does not speak to the potentially different theoretically optimal emissions control rates across different GHGs or to different cost functions. The results for base emissions are shown in Table G-1.

[15] For a further discussion, see *Background Notes on non-CO_2 Forcings* (47).

Item	2020	2025	2050	2100
Base/NC industrial emissions (GtCO2/yr)	39.55	43.38	61.12	78.54
Base/NC land emissions (GtCO2/yr)	5.90	5.31	3.14	1.09
Abateable nonCO2 emissions GHG (GtCO2e/yr)	9.24	9.56	11.14	14.30
**Total, CO2-e abateable emissions (GtCO2e/yr)**	**54.69**	**58.24**	**75.39**	**93.93**
Base/NC CO2 forcings (W/m2)	2.43	2.69	4.04	6.62
Abateable other forcings (w/m2)	1.16	1.10	1.04	1.28
Exogenous forcings (w/m2)	(0.20)	(0.16)	0.04	0.44
**Total abateable forcings (w/m2)**	**3.39**	**3.63**	**5.12**	**8.34**

Base/NC = with zero emissions control

Table G-1. Emissions and forcings for different GHGs

To implement the inclusion of the new gases, we first convert industrial CO_2 emissions to total CO_2-equivalent emissions. This is accomplished by assuming that the relationship is a simple trend. This is written as follows in GAMS language:

(G.1) sigmatot(t)=sigma(t)*(1+0.149*exp((-.167)*(t.val-1)));

The variable "sigmatot(t)" is the ratio of total CO_2-e emissions to output – that is, including both CO_2 and abatable non-CO_2 emissions - while "sigma(t)" is the ratio of industrial emissions to output. Eq (G.1) assumes that total is initially 1.149 times industrial CO_2 emissions and declines at 0.167 per period. The average error for the approximation is 0.17% for 2020-2100.

Equation (G.1) implies a trajectory of base (no controls) CO_2-e emissions from non- CO_2 GHGs, $ECO2e_{NonCO2GHGabate}(t)$. Realized non-$CO_2$ GHGs in CO_2-e units are then given by base emissions times one minus the emissions control rate (see equation (6) in the paper). The increased forcings resulting from these emissions, $F_{ABATE}(t)$, which add to the total change in radiative forcings (see equation (12) in the paper), are in turn given by:

(G.2) $F_{ABATE}(t+1) = Fcoef2 * F_{ABATE}(t) + Fcoef1 * ECO2e_{NonCO2GHGabate}(t)(1-\mu(t))$

where *Fcoef2* and *Fcoef1* are parameters reflecting the persistence and initial emissions-forcings impact, respectively.

After all the changes, the abatable emissions are 95% of total anthropogenic forcings compared to 80% of forcings in the DICE-2016 model. A full discussion is in a note "abatement_nonco2ghg.docx," with associated Excel sheet, "non-co2GHG-MAC.xlsx."

Appendix H. Sources for data and concepts for DICE-2023

H.1. Global Output

Estimates are from IMF and World Bank. For 2019, the two sources give virtually identical estimates for PPP in current international dollars. For the IMF, GDP in 2019 in PPP current international dollars = $134,916 billion (as of October 2021). Historical series are from both IMF and World Bank from 1980 to 2021, and then spliced from 1991 backwards with older series because the data before 1990 omit former USSR countries such as Russia. Table H-1 shows the growth rate history and projections. We assume that GDP is back to trend by 2026. Figure H-1 shows the ratio of World Bank to IMF real PPP GDP.

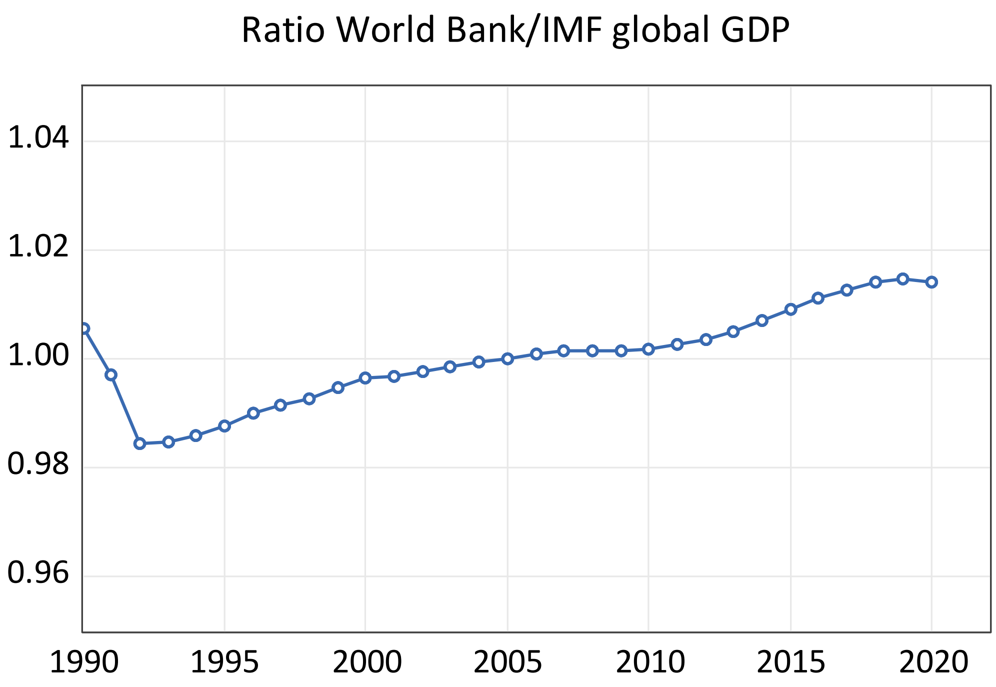

Figure H-1. Ratio of World Bank to IMF global real output

	Real GDP World Bank	Real GDP IMF	Population, World Bank	p.c. GDP World Bank	p.c. GDP IMF
	Billions of 2014 US international $, PPP			2014 US international $, PPP	
1980		38,861	4,433		8,766
1990	50,828	52,134	5,280	9,626	9,874
2000	67,549	69,927	6,114	11,048	11,437
2010	95,833	98,674	6,922	13,845	14,255
2019	129,427	131,541	7,673	16,867	17,143
2026		160,333	8,216		19,514
	Growth rate (%/year)				
1980-90		2.98%	1.76%		1.20%
1990-2000	2.88%	2.98%	1.48%	1.39%	1.48%
2000-10	3.56%	3.50%	1.25%	2.28%	2.23%
2010-20	3.40%	3.25%	1.15%	2.22%	2.07%
2019-26		2.87%	0.98%		1.87%
2000 - 2019	3.48%	3.38%	1.20%	2.25%	2.15%
2019 - 2026		2.87%	0.98%		1.87%
Pre- to post-pandemic		0.51%	0.22%		0.28%

Table H-1. Rate of growth of global real GDP, Population, per capita real GDP, history, and projections
Sources: IMF, World Bank, and UN.

H.2. Population

Historical data for population are from the World Bank, while future projections are from the UN. Note that UN projections tend to be high relative to other projections, but the UN has the most detailed data and is continuously revised. Figure H-2 shows logarithmic growth rates.

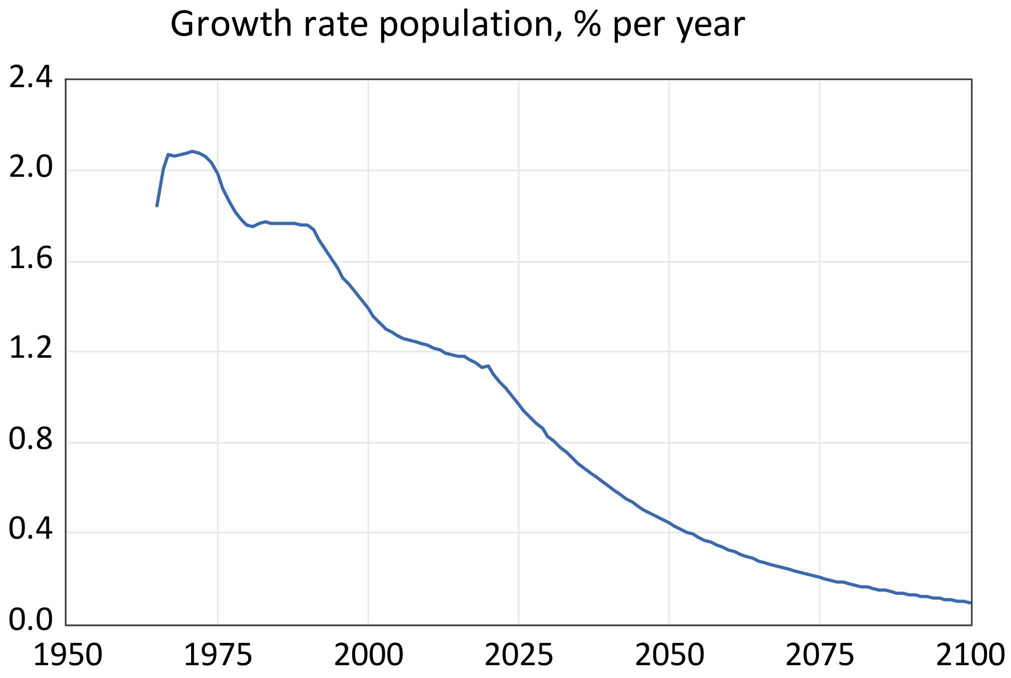

Figure H-2. Rate of growth of global population: history to 2020 and UN projection to 2100
Source: UN.

H3. CO_2 emissions and the emissions-output ratio for industrial CO_2

Industrial CO_2 emissions are from EDGAR from 1970 and then linked to CDIAC from 1765 to 1970. Note that CDIAC estimates of CO_2 emissions are about 10% higher than EDGAR for historical data (pre-2015).

Calculated values of the ratio of industrial CO_2 emissions to real GDP ("sigma" or σ in our notation) is calculated from the above series on emissions and GDP. Figure H-3 shows the 5-year moving average of the decarbonization rate. Figure H-4 shows the actual values and trend of sigma (CO_2/output), with the trend calculated since 1970. Earlier analysis suggests that the recent increase in the rate of decarbonization is primarily due to China. Nonetheless, we take global estimates and assume that the new baseline of the rate of change of σ is -1.5% per year. We assume that -½% point per year of this decline is due to policy in our new estimates. That is, we assume 5% abatement in 2020 relative to no-policy.

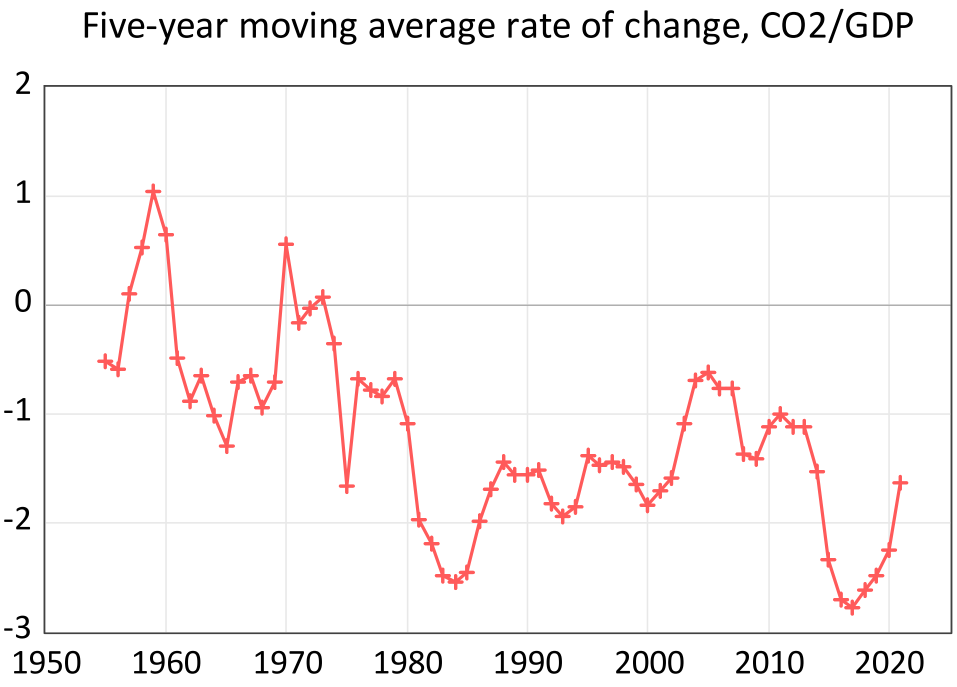

Figure H-3. Moving average rate of growth of industrial CO_2 emissions/GDP

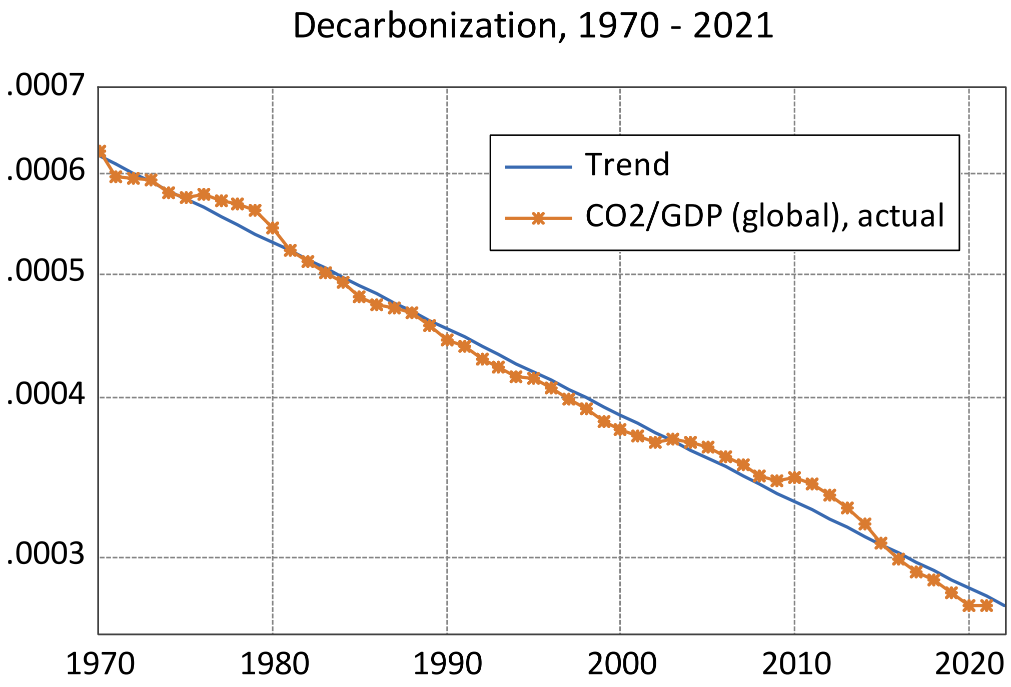

Figure H-4. History and trend of global ratio, emissions/GDP, 1970 - 2021

Appendix I. Baseline emissions and carbon prices

Projecting baseline emissions has two separate issues. One is baseline climate policy and the second is the baseline rate of decarbonization.

Current policy is reasonably well-measured for carbon prices, which are \$3/t CO_2 in 2021 according to the World Bank. However, this is complicated by the presence of other energy taxes and subsidies as well as regulatory equivalents of energy taxes other than those aimed at climate policy (such as fuel taxes). Energy subsidies are in the order of \$10/t CO_2 according to estimates of the OECD. The impact of non-carbon taxes and energy and climate regulations are poorly estimated at this time.

For present purposes, we assume that non-carbon taxes offset subsidies and that regulatory measures are about the equivalent of explicit carbon taxes, putting total effective taxes at \$6/tCO_2 in 2020.

We then calculate baseline emissions intensity as the ratio of estimated CO_2-output ratio divided by $[1-\mu(t)]$, where $\mu(t)=$ the emissions control rate. The implicit carbon price is estimated from the abatement function. For 2020, these yields a $\mu_t = 5\%$ and a carbon price of \$6/tCO_2.

Appendix J. Projections of per capita GDP growth[16]

Projections of future growth of per capita output and consumption have been revised in October 2023 in light of recent data and research. The results relative to earlier models and versions yield a slightly lower growth rate in the early years (to 2050), but more rapid growth in per capita global output over the model horizon. Note that the growth rates after 2150 make little difference in scenarios with strong policies, but they can affect the base (current policy) results. The reason is that most strong policies have virtually 100% emissions control after a century, so growth projections after that will have little to no effect on emissions, concentrations, and temperature.

Historical data and current projections are shown in Table J-1. The DICE-2023 projections in the last column are intermediate between the CGN (8)/MSW (9) results and the Rennert et al. (10) blended statistical and expert elicitations.

	Historical data (geometric mean, percent per year)						
	MSW	IMF	CGN	MSW	Renn-med	Renn-avg	DICE-2023
1901-1931	1.3						
1931 - 1960	1.9						
1961 - 1990	2.1	2.3					
1991 - 2022		2.0					
	Projections (geometric mean, percent per year)						
2020 - 2050			2.6	1.9	1.5	1.5	1.9
2020 - 2100			2.0	1.9	1.5	1.5	1.8
2020 - 2200				1.9	0.9	1.1	1.7
2020 - 2300				1.9	0.9	1.1	1.6

MSW = Muller, Stock, and Watson
IMF = International Monetary Fund from historical data base
CGN = Christensen, Gillingham, and Nordhaus
MSW = Muller, Stock, and Watson
Renn-med= Rennert et al., Figure 6 (from background data)
Renn-avg = Rennert et al., Figure 6 (from our calculations)
DICE-2023 = from base run of version b-4-3-6

Table J-1. Estimates of the growth in per capita output from different studies.

A key parameter for calculating the precautionary effect on discounting is the uncertainty about future growth. For this parameter, we examined the dispersion of forecasts. Standard deviations or quantiles of the distributions of growth rates were tabulated in different studies, and these yielded the estimated standard deviations of trend growth in per capita global output shown in Table J-2.

[16] Note that this Appendix was completely revised in October 2023.

	Estimated standard deviation of trend growth (% points/year)				
	CGN	MSW	NPP	Renn	DICE-2023
2020 - 2050	1.1	1.0	1.0	0.9	1.0
2020 - 2100	1.1	1.0	0.8	0.8	1.0
2020 - 2200			0.8	0.7	1.0
2020 - 2300			0.9	0.6	1.0

CGN = Christensen, Gillingham, and Nordhaus
MSW = Muller, Stock, and Watson
NPP = Newell, Pizer, Prest
Renn = Rennert et al., Figure 6, calculated from (5,95) percentiles.
DICE-2023 = from base run of version b-4-3-6

Table J-2. Estimates of the uncertainty of the growth in per capita output from different studies. Note that these are the uncertainty about the *trend* rate of growth over the periods. As a simple illustration, if half the growth paths have a rate of growth of 3% per year to 2100 and half the paths have a rate of growth of 1% per year, then the standard deviation would be 1% point per year, as is assumed for the DICE model.

For modeling purposes, we choose a constant uncertainty of 1.0% point per year because of the simplicity of modeling and transparency of interpretation. This rate is close to the near-term uncertainty for most estimates but lower than estimates after 2100. Since the precautionary impact is proportional to the variance times the squared time-from-present, this assumption will tend to overestimate the precautionary impact after 2100.

The *Background Notes on Discounting* (13) provides additional comparisons and the tables and figures from which the estimates in this appendix are drawn.

Appendix K. Computational and algorithmic aspects

IAMs are computationally complex compared to physical science models, such as climate models, which use recursive time-stepped algorithms. The DICE model is a nonlinear optimization problem with nonlinear inequality and equality constraints. The model is usually solved using the CONOPT or NLP solver in the GAMS modeling system (48). This is based on the generalized reduced gradient (GRG) algorithm. The basic approach is to embed a linear programming algorithm inside an algorithm that linearizes the nonlinear equations. While this algorithm does not guarantee that the solution is the global optimum, our experience over the years has not suggested any solutions other than those found by the algorithm. The model can also be run using EXCEL Solver (most conveniently using the Risk Solver Platform or other premium products). Using EXCEL Solver is also much easier to understand and detect programming errors. DICE-2023 has not yet been implemented, but we expect to do so soon. For the standard run for 400 years and 11 scenarios, the execution time in GAMS is 10 seconds on a high-end 2021 PC.

One of the unfortunate byproducts of greater attention to the details of various sectors, such as the carbon cycle, is the increased complexity of the DICE model over the years. The 2023 version has about 2½ times more variables and equations than the 1992 version. However, the 1992 version, code, and computers took 180 times longer than the 2023 version, code, and computers (the 1992 experience is reported in (49)). Computers and software have vastly outpaced modeling, but whether that has improved accuracy will await a few more decades of experience.

The code and a description of the model are available at https://bit.ly/3TwJ5nO.

Appendix L. Gams source code

The GAMS source code for the current version of DICE-2023 is available in the folder "DICE-2023-GAMS-4-3-10" on the Box web site https://bit.ly/3TwJ5nO.

It is also available on Nordhaus's website at williamnordhaus.com under the header "New: DICE-2023 model."

Note that the current version requires GAMS software to operate, and the full GAMS version is proprietary. A trial version is free, and an academic license can be purchased for $320 at https://www.gams.com/sales/pricing_academic/.

An Excel version is available at the BOX site, https://bit.ly/3TwJ5nO. Note that the Excel version does not track the authoritative GAMS version exactly. Versions in other software systems are being prepared and will be available at the BOX site soon.

Appendix M. References for Main Text and Appendix

(1) Millar, Richard J., Zebedee R. Nicholls , Pierre Friedlingstein, and Myles R. Allen. 2017. "A modified impulse-response representation of the global near-surface air temperature and atmospheric concentration response to carbon dioxide emissions," *Atmos. Chem. Phys.,* 17, 7213–7228.

(2) IPCC AR6. 2021. Masson-Delmotte, Valérie, Panmao Zhai, Anna Pirani, et al., *Climate change 2021: the physical science basis." Contribution of working group I to the sixth assessment report of the intergovernmental panel on climate change*: 2.

(3) Dietz, Simon, Christian Gollier, and Louise Kessler. 2018. "The climate beta*." Journal of Environmental Economics and Management* 87: 258-274.

(4) Nordhaus, William D., and Andrew Moffat. 2017. "A Survey of Global Impacts of Climate Change: Replication, Survey Methods, and a Statistical Analysis." *NBER Working Papers* 23646.

(5) Piontek, Franziska, Laurent Drouet, Johannes Emmerling, Tom Kompas, Aurélie Méjean, Christian Otto, James Rising, Bjoern Soergel, Nicolas Taconet, and Massimo Tavoni. 2021. "Integrated perspective on translating biophysical to economic impacts of climate change." *Nature Climate Change* 11, no. 7: 563-572.

(6) Dietz, Simon, James Rising, Thomas Stoerk, and Gernot Wagner. "Economic impacts of tipping points in the climate system," *PNAS* (24 August 2021). doi: 10.1073/pnas.2103081118

(7) Harmsen, J. H. M., Detlef P. van Vuuren, Dali R. Nayak, Andries F. Hof, Lena Höglund-Isaksson, Paul L. Lucas, Jens B. Nielsen, Pete Smith, and Elke Stehfest. 2019. "Long-term marginal abatement cost curves of non-CO_2 greenhouse gases." *Environmental Science and Policy* 99: 136-149.

(8) Christensen, Peter, Kenneth Gillingham, and William Nordhaus. 2018. "Uncertainty in forecasts of long-run economic growth." *Proceedings of the National Academy of Sciences* 115, no. 21: 5409-5414.

(9) Müller, Ulrich K., James H. Stock, and Mark W. Watson. "An econometric model of international growth dynamics for long-horizon forecasting." Review of Economics and Statistics 104, no. 5 (2022): 857-876.

(10) Rennert, Kevin, Brian C. Prest, William A. Pizer, Richard G. Newell, David Anthoff, Cora Kingdon, Lisa Rennels et al. "The social cost of carbon: advances in long-term probabilistic projections of population, GDP, emissions, and discount rates." *Brookings Papers on Economic Activity 2021*, no. 2 (2021): 223-305.

(11) Newell, Richard G., William A. Pizer, and Brian C. Prest. "A discounting rule for the social cost of carbon." Journal of the Association of Environmental and Resource Economists 9, no. 5 (2022): 1017-1046.

(12) Riahi, K. et al. 2021. Long-term economic benefits of stabilizing warming without overshoot – The ENGAGE model intercomparison. *Res. Sq.* [Preprint]. 10.21203/rs.3.rs-127847/v1.

(13) *Background Notes on Discounting*, available at https://bit.ly/3TwJ5nO in the folder “DICE Folders/Background Papers for DICE 2023.”

(14) Gollier, Christian. "Evaluation of long-dated assets: The role of parameter uncertainty." *Journal of Monetary Economics* 84 (2016): 66-83.

(15) NAS. 2017. National Academies of Sciences, Engineering, and Medicine. *Valuing Climate Damages: Updating Estimation of the Social Cost of Carbon Dioxide*. Washington, DC: The National Academies Press. https://doi.org/10.17226/24651.

(16) Prest, Brian. “Disentangling the Roles of Growth Uncertainty, Discounting, and the Climate Beta on the Social Cost of Carbon” *RFF Working Paper 23-41.*

(17) Revelle, R. and Suess, H. E. 1957. “Carbon dioxide exchange between atmosphere and ocean and the question of an increase of atmospheric CO_2 during the past decades,” *Tellus*, 9, 18–27.

(18) Joos, F., Roth, R., Fuglestvedt, J. S., Peters, et al. 2013. “Carbon dioxide and climate impulse response functions for the computation of greenhouse gas metrics: a multimodel analysis.” *Atmospheric Chemistry and Physics,* 13, 5, 2793-2825.

(19) Dietz, Simon, Frederick van der Ploeg, Armon Rezai, Frank Venmans. 2021a. “Are Economists Getting Climate Dynamics Right and Does It Matter?” *Journal of the Association of Environmental and Resource Economists*, 8(5). DOI: https://doi.org/10.1086/713977.

(20) *Background Notes on DFAIR*, available at https://bit.ly/3TwJ5nO in the folder “DICE Folders/Background Papers for DICE 2023.”

(21) Riahi, K. et al, 2021a. Long-term economic benefits of stabilizing warming without overshoot – The ENGAGE model intercomparison. Data provided by authors, 2021.

(22) *Background Notes on Damages,* available at https://bit.ly/3TwJ5nO in the folder “DICE Folders/Background Papers for DICE 2023.”

(23) Dellink, Rob, Elisa Lanzi, and Jean Chateau. 2019. “The sectoral and regional economic consequences of climate change to 2060.” *Environmental and Resource Economics* 72, 309–363.

(24) Roson, Roberto, and Martina Sartori. 2016. “Estimation of climate change damage functions for 140 regions in the GTAP9 database.” *Journal of Global Economic Analysis,* 1(2): 78-115.

(25) Takakura, Jun'ya, Shinichiro Fujimori, Naota Hanasaki, Tomoko Hasegawa, Yukiko Hirabayashi, Yasushi Honda, Toshichika Iizumi et al. 2019. "Dependence of economic impacts of climate change on anthropogenically directed pathways." *Nature Climate Change* 9, no. 10: 737-741.

(26) Kompas, Tom, Van Ha Pham, and Tuong Nhu Che. 2018. "The effects of climate change on GDP by country and the global economic gains from complying with the Paris climate accord." *Earth's Future* 6, no. 8: 1153-1173.

(27) Zhao, Zi-Jian, Xiao-Tong Chen, Chang-Yi Liu, Fang Yang, Xin Tan, Yang Zhao, Han Huang et al. 2020."Global climate damage in 2° C and 1.5° C scenarios based on BCC_SESM model in IAM framework." *Advances in Climate Change Research* 11, no. 3: 261-272.

(28) Letta, Marco, and Richard SJ Tol. 2019. "Weather, climate and total factor productivity." *Environmental and Resource Economics* 73, no. 1: 283-305.

(29) Burke, Marshall, Solomon M. Hsiang, and Edward Miguel. 2015. "Global nonlinear effect of temperature on economic production." *Nature* 527, no. 7577: 235-239.

(30) Newell, Richard G., Brian C. Prest, and Steven E. Sexton. 2021. "The GDP-temperature relationship: implications for climate change damages." *Journal of Environmental Economics and Management* 108: 102445.

(31) Pretis, Felix, Moritz Schwarz, Kevin Tang, Karsten Haustein, and Myles R. Allen. 2018. "Uncertain impacts on economic growth when stabilizing global temperatures at 1.5 C or 2 C warming." *Philosophical Transactions of the Royal Society A: Mathematical, Physical and Engineering Sciences* 376, no. 2119: 20160460.

(32) Kahn, Matthew E., Kamiar Mohaddes, Ryan NC Ng, M. Hashem Pesaran, Mehdi Raissi, and Jui-Chung Yang. 2019. "Long-term macroeconomic effects of climate change: A cross-country analysis." *Energy Economics*, 104:105624.

(33) Kalkuhl, Matthias, and Leonie Wenz. 2020. "The impact of climate conditions on economic production. Evidence from a global panel of regions." *Journal of Environmental Economics and Management* 103: 102360.

(34) Cline, William R. 1992. *The economics of global warming.* Institute for International Economics, Washington, DC: 399.

(35) Dellink, Rob, Ellisa Lanzi, Jean Chateau, Francesco Bosello, Ramiro Parrado, and Kelly De Bruin. 2014. "Consequences of climate change damages for economic growth: a dynamic quantitative assessment." *OECD Working Paper 1135.*

(36) Bosello, Francesco, Fabio Eboli, and Roberta Pierfederici. 2012. "Assessing the economic impacts of climate change." FEEM (Fondazione Eni Enrico Mattei), Review of Environment, Energy and Economics (Re3).

(37) Nordhaus, William, and Paul Sztorc. 2013. *DICE 2013R: Introduction and user's manual.* Yale University and the National Bureau of Economic Research, USA.

(38) Gillingham, K. et al., 2018. Modeling uncertainty in integrated assessment of climate change: A multimodel comparison. *J. Assoc. Environ. Resour. Econ.* 5, 791–826.
Gollier, Christian. 2013. *Pricing the planet's future: the economics of discounting in an uncertain world.* Princeton University Press.

(39) Nordhaus, William. 2018. "Projections and uncertainties about climate change in an era of minimal climate policies." *American Economic Journal: Economic Policy* 10, no. 3: 333-60.

(40) Cai, Yongyang, and Thomas S. Lontzek. "The social cost of carbon with economic and climate risks." *Journal of Political Economy* 127, no. 6 (2019): 2684-2734.

(41) Barnett, Michael, William Brock, and Lars Peter Hansen. 2020. "Pricing uncertainty induced by climate change." *The Review of Financial Studies* 33, no. 3: 1024-1066.

(42) van den Bremer, Ton S., and Frederick Van der Ploeg. 2021. "The risk-adjusted carbon price." *American Economic Review* 111, no. 9: 2782-2810.

(43) Lemoine, Derek. 2021. "The climate risk premium: how uncertainty affects the social cost of carbon." *Journal of the Association of Environmental and Resource Economists* 8, no. 1: 27-57.

(44) Carleton, Tamma, Amir Jina, Michael Delgado, Michael Greenstone, Trevor Houser, Solomon Hsiang, Andrew Hultgren, et al. 2022. "Valuing the global mortality consequences of climate change accounting for adaptation costs and benefits." *The Quarterly Journal of Economics* 137, no. 4: 2037-2105.

(45) Rode, Ashwin, Tamma Carleton, Michael Delgado, Michael Greenstone, Trevor Houser, Solomon Hsiang, Andrew Hultgren, et al. 2021. "Estimating a social cost of carbon for global energy consumption." *Nature* 598, no. 7880: 308-314.

(46) Howard, Peter H., and Thomas Sterner. 2017. "Few and not so far between: a meta-analysis of climate damage estimates." *Environmental and Resource Economics* 68, no. 1: 197-225.

(47) *Background Notes on non-CO2 Forcings,* available at https://bit.ly/3TwJ5nO in the folder "DICE Folders/Background Papers for DICE 2023."

(48) Brooke, Anthony, David Kendrick, Alexander Meeraus, and Ramesh Raman (2005). *GAMS: A Users Guide,* GAMS Development Corporation, Washington, D.C.

(49) Nordhaus, William D. 1992. "GAMS program for DICE Model," Cowles Foundation Note, December 10 in "modecode.d1."
